# Evaluation of bony fusion after posterior lumbar interbody fusion: a systematic review and meta-analysis

**DOI:** 10.1016/j.bas.2026.106067

**Published:** 2026-04-22

**Authors:** Luc W.F. van Haaster, Rania A. Mekary, Carmen L.A. Vleggeert-Lankamp

**Affiliations:** a Department of Neurosurgery, Leiden University Medical Center, Leiden, Albinusdreef 2, Leiden, 2333 ZA, the Netherlands; b Department of Pharmaceutical Business and Administrative Sciences, School of Pharmacy, MCPHS University, 179, Longwood Ave, Boston, MA, 02115, United States of America; c Computational Neuroscience Outcomes Center at Harvard, Department of Neurosurgery, Brigham and Women's Hospital, 75 Francis St, Boston, MA, 02115, United States of America; d Department of Neurosurgery, Spaarne Gasthuis Hospital, Spaarnepoort 1, Hoofddorp, 2134 TM, the Netherlands

**Keywords:** Bony fusion, Fusion rate, Lumbar spine, Posterior lumbar interbody fusion, Dynamic X-ray

## Abstract

**Introduction:**

Posterior lumbar interbody fusion (PLIF) is a common surgical procedure for degenerative lumbar conditions, aiming to decompress neural tissue and stabilize the spine. Bony fusion is essential for long-term success, but inconsistent radiological criteria lead to variable reported fusion rates. This systematic review and meta-analysis evaluated quantitative criteria for assessing bony fusion and their impact on fusion rates over time.

**Research question:**

What quantitative criteria are used to assess bony fusion after PLIF, and how do these criteria affect fusion rates at different postoperative time points?

**Material and methods:**

PubMed, MEDLINE, Embase, Web of Science, Cochrane Library, and CINAHL were searched through November 6, 2025, for studies assessing bony fusion after PLIF using quantitative criteria on dynamic X-ray or CT. Pooled fusion rates were calculated at different postoperative time points, including additional analyses for cage material, smoking, and segmental motion cut-offs.

**Results:**

Seventeen studies including 1390 patients were analyzed. Fusion was assessed quantitatively exclusively by dynamic X-ray. Pooled fusion rates increased over time: 76.0% at 6 months, 86.4% at 12 months, 90.2% at 24 months, and 93.4% at 48 months. Optimal cut-off values for segmental motion of ≤3° and ≤4° were chosen due to similar heterogeneity, which was the lowest at 24 months.

**Discussion and conclusion:**

Fusion rates reach approximately 90% 24 months after PLIF and show minimal increase thereafter. Evaluating segmental motion with a ≤3°- or ≤4° cut-off provides the most reliable assessment of bony fusion.

## Highlights

• Fusion rates after PLIF increase from 76.0% at 6 months, to 86.4% at 12 months, 90.2% at 24 months, and 93.4% at 48 months.
• A cut-off of ≤3° or ≤4° in segmental motion during flexion/extension reliably assesses bony fusion.
• There was no meaningful difference in fusion rates between titanium and PEEK cage materials.
• Heterogeneity was most favorable at 24 months, and was not significantly different using the ≤3°- or ≤4° cut-off value.

## 1 Introduction

Posterior lumbar interbody fusion (PLIF) is a surgical procedure that is increasingly used for the treatment of degenerative spinal conditions, including spinal stenosis with accompanying spondylolisthesis, radiculopathy due to (recurrent) disc herniation, and clinically relevant foraminal stenosis (Mobbs et al., 2015; Spiker et al., 2019). PLIF is carried out by performing bilateral facetectomy, laminectomy, and insertion of pedicle screws, followed by discectomy and placement of bilateral intervertebral cages, optionally packed with autologous bone or demineralized bone matrix (Mobbs et al., 2015; Reid et al., 2019). The goal of PLIF surgery is to alleviate pain and neurological symptoms by decompressing nervous tissue and, at the same time, restoring disc height and providing stability to the operated segment, thereby allowing intervertebral bony fusion to occur (Mobbs et al., 2015; Spiker et al., 2019; Reid et al., 2019). Clinically relevant technique variability exists between procedures, specifically regarding the type of screw trajectory (pedicle versus cortical bone), interbody cage material, and the specific graft substrates used to fill the cage and pack the intervertebral space (Sakaura et al., 2018; Meng et al., 2021). In the post-operative period, clinical improvement can be evaluated by questioning and examining the patient, but evaluation of radiological fusion is still a matter of debate. The absence of fusion can have clinical consequences in the long-term as this increases the risk of implant failure and revision surgery (Lee et al., 2004) and is therefore considered an important parameter.

While minor differences in surgical techniques and materials contribute to the variability of fusion rates to some extent, the main reason is the lack of a consistent radiological definition of bony fusion (Formica et al., 2020). Several qualitative measures have been described in literature (Yu et al., 2025a; Duits et al., 2024), but this subjective evaluation method is presumably the cause of marked variability in reported fusion rates, underscoring the need for a uniform and quantifiable definition of bony fusion. Therefore, the primary objective of this systematic review and meta-analysis was to give an overview of bony fusion rates after PLIF surgery based on quantitative measurement methods. When deemed possible, the secondary objective was to evaluate the results for various cage materials and to perform sensitivity analyses of the literature data to evaluate whether there was an optimal cut-off value to serve as a discriminant parameter for fusion to be present. This resulted in the following research question: ‘‘What are the pooled bony fusion rates at different postoperative timepoints following PLIF surgery when assessed using a quantitative fusion criterion, compared across different cut-off thresholds and different interbody cage materials?’‘.

## 2 Material and methods

### 2.1 Search methods for the identification of studies

The PRISMA 2020 27-item checklist was utilized as the reporting framework for this review (Page et al., 2021). A comprehensive search was performed across six medical literature databases: PubMed, MEDLINE, Embase, Web of Science, Cochrane Library, and CINAHL. The search spanned from inception to November 6, 2025 (Appendix A).

### 2.2 Criteria for considering studies for this review

Two authors (LWFH and CLAVL) screened all abstracts and titles independently for eligibility in this systematic review and meta-analysis, based on pre-defined criteria. The inclusion criteria required the article to meet the following conditions: the article had to be an original report presenting primary data and to be published in English or Dutch. In addition, the study required at least 20 patients to undergo a 1- or 2-level posterior lumbar instrumented fusion with bilateral cages (interbody fusion) of the lumbar spine (Th12-S1). The nature of the material of which the cage is made had to be evident, but it could not be biodegradable. Furthermore, bony fusion had to be assessed quantitatively with dynamic X-ray or computed tomography, and the method of assessing bony fusion had to be described. Finally, a minimum follow-up duration of three months was required, and the article had to be published in a peer-reviewed journal. According to the exclusion criteria, studies containing patients with more than 2-segment stabilization, patients with lumbar fusion in response to a traumatic lesion or tumor, and patients receiving bone inductive treatment (e.g., bone morphogenetic protein or teriparatide) were not eligible for inclusion.

The same authors (LWFH and CLAVL) independently completed the full-text screening of the remaining records, according to the aforementioned in- and exclusion criteria. Possible discrepancies were discussed and agreed upon.

### 2.3 Assessment of risk-of-bias for the included studies

The quality of the included articles was assessed independently by two authors (LWFH and CLAVL), based on an adjusted version of the Dutch Cochrane Centre checklist for cohort studies using a method adapted from Furlan. et al. (Furlan et al., 2009) (Appendix B). In case an article presented two or more treatment arms, these were treated as separate case series, as the focus of this review was on evaluating fusion rates within each treatment arm, as opposed to comparing the different surgical interventions. All included articles were scored based on three domains: selection bias, outcome bias, and follow-up bias. Three points could be awarded per domain, contributing to a maximum total score of 9 points. A distinction was made between studies with 6 points or more, which were classified as ‘‘low risk of bias'’ and studies with 5 points or less, which were classified as ‘‘high risk of bias'’. Possible disagreements were discussed and agreed upon.

### 2.4 Data collection and analysis

Data were extracted and entered in an Excel table independently by two authors and included: author, title, publication year, study design, demographic information of study participants, smoking, screw trajectory (pedicle screws or cortical bone trajectory screws), cage type and material, use of graft substrate and fusion parameters such as fusion level, number of fusions, fusion assessment and fusion rates at 3, 6, 12, 24 and 48 months postoperatively. All extracted data were transferred to the Comprehensive Meta-Analysis version 4 software for data analysis. q (DerSimonian and Laird, 1986). Subgroup analyses according to cage type were performed at all timepoints. Stratified analyses by study quality were performed when studies of both high and low quality were included. The Cochrane Q statistics with a significance level of p < 0.10 was used to evaluate the presence of heterogeneity (Huedo-Medina et al., 2006). Heterogeneity was quantified using the I-squared (I^2^) index, reported as low (0-25%), moderate (26-50%), substantial (51-75%), or high (76-100%). Begg's test was used to detect any potential asymmetry observed in the funnel plot due to the small-study effect. In case of any asymmetry in the funnel plot proven visually or statistically, and when the heterogeneity was low-to moderate, the trim-and-fill method was used to impute potentially missing records and calculate the adjusted pooled fusion rates, contingent on publication bias being the source of asymmetry. Sensitivity analyses were carried out to demonstrate potential differences in fusion rates as a result of different cut-off values for descriptive criteria. Meta-regression was performed when at least ten studies were available at a given timepoint and was utilized to evaluate the statistical relationship between smoking and fusion rates. A two-sided p-value <0.05 was considered statistically significant, unless specified otherwise.

## 3 Results

### 3.1 Search and selection results

**Fig. 1:**
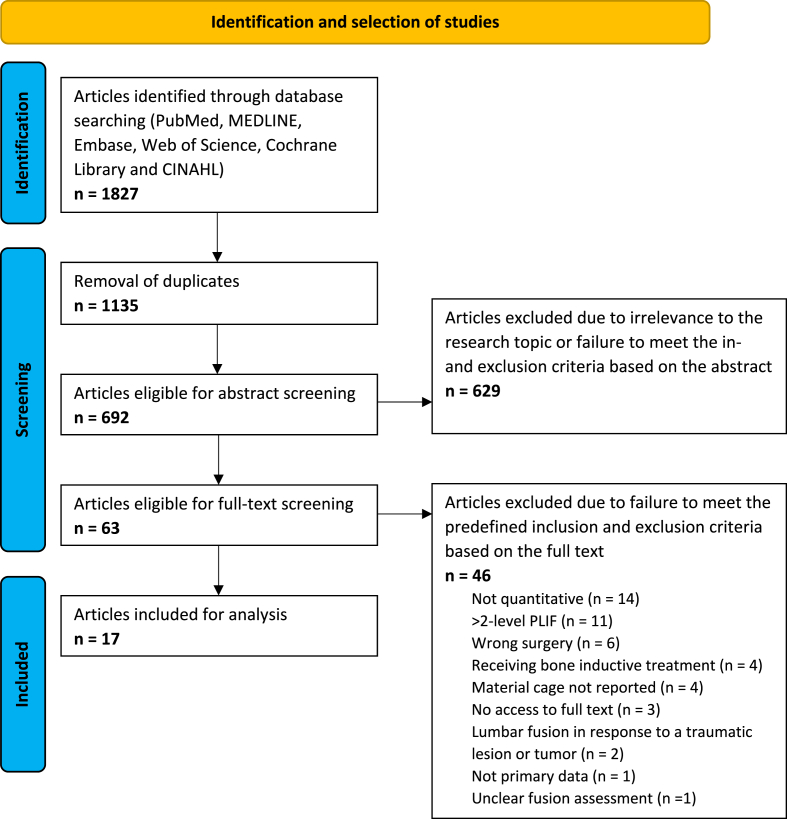
Flow diagram of the identification and selection of studies.

The initial search identified 1827 records. After removal of 1135 duplicates, 692 unique articles remained for title and abstract screening. Of these, 629 were excluded as they were not relevant to the research question or did not meet the predefined eligibility criteria based on abstract review. Full‐text screening was subsequently conducted for the remaining 63 articles. Following full‐text assessment, 46 articles were excluded. The resulting 17 studies were included in the analysis (Yamagishi et al., 2024; Ham et al., 2023, 2024; Yanai et al., 2020; Makino et al., 2021; Schnake et al., 2021; Lebhar et al., 2020; Lee et al., 2015, 2016, 2017, 2018, 2020; Konomi et al., 2020; Lin et al., 2016; Han et al., 2015; Lee and Lee, 2015; Kim et al., 2006) (Fig. 1).

The 17 analyzed articles included 1390 patients spread over six randomized controlled trials (n = 386), seven retrospective cohort studies (n = 700), and four prospective cohort studies (n = 304). As mentioned before, in case an article reported two or more treatment arms, these would be treated as separate case series, resulting in a total of 30 case series out of 17 articles. Five studies, comprising nine case series, reported fusion rates at six months (Makino et al., 2021; Lee et al., 2015, 2016, 2018; Kim et al., 2006). Fifteen studies, consisting of 26 case series, reported fusion rates at 12 months (Yamagishi et al., 2024; Ham et al., 2023, 2024; Makino et al., 2021; Schnake et al., 2021; Lebhar et al., 2020; Konomi et al., 2020; Lee et al., 2015, 2016, 2017, 2018; Lin et al., 2016; Han et al., 2015; Lee and Lee, 2015; Kim et al., 2006). Six studies, including 11 case series, reported fusion rates at 24 months (Yamagishi et al., 2024; Yanai et al., 2020; Konomi et al., 2020; Lin et al., 2016; Lee and Lee, 2015; Lee et al., 2017). One study, comprising two case series, reported fusion rates at 48 months (Table 1) (Lee et al., 2020).

**Table 1:** Overview of included articles.

Author and year	Follow-up (months)	Cage type + graft substrate + screw trajectory	Fusion level | number of fusions	Fusion assessment criteria	Fusion rate (%) | follow-up time	Smokers (%)	Number of patients (mean age)	Risk of bias
Yamagishi A, 2024a	12, 24	Titanium-coated PEEK cages + autologous bone graft + cortical bone trajectory screws	L3-L4 | 11 L4-L5 | 51 L5-S1 |6	≤5° segmental motion on lateral dynamic radiographs, the presence of bridging bone, and the absence of pedicle screw loosening	86.8% | 12 months 89.7% | 24 months	-	68 (65.3)	7 (low)
Yamagishi A, 2024b	12, 24	Carbon fiber-reinforced PEEK cages + autologous bone graft + cortical bone trajectory screws	L1-L2 | 1 L2-L3 | 1 L3-L4 | 14 L4-L5 | 68 L5-L6 | 1 L5-S1 | 3	≤5° segmental motion on lateral dynamic radiographs, the presence of bridging bone and the absence of pedicle screw loosening	77.5% | 12 months 88.8% | 24 months	-	89 (68.5)	7 (low)
Ham DW, 2023a	12	Titanium cages (1 window, 1 non-window) + autologous bone graft (window cage) + pedicle screws	L2-L3 | 1 L4-L5 | 14 L5-S1 | 16	≤3° segmental motion and no cage subsidence on lateral dynamic radiographs	87.1% | 12 months	-	31 (74.2)	6 (low)
Yanai Y, 2020a	24	PEEK cages + cortical bone trajectory screws	L2-L3 | 1∗∗ L3-L4 | 4∗∗ L4-L5 | 50∗∗ L5-S1 | 14∗∗	≤3° segmental motion on lateral dynamic radiographs, the presence of bridging bone, no translucency around the cages, and thick fusion mass formation	83.8% | 24 months	-	37 (51.1)	5 (high)
Yanai Y, 2020b	24	Titanium cages + cortical bone trajectory screws	L2-L3 | 1∗∗ L3-L4 | 4∗∗ L4-L5 | 50∗∗ L5-S1 | 14∗∗	≤3° segmental motion on lateral dynamic radiographs, the presence of bridging bone, no translucency around the cages, and thick fusion mass formation	93.8% | 24 months	-	32 (51.1)	5 (high)
Makino T, 2021a	6, 12	Titanium-coated PEEK cages + autologous bone graft + pedicle screws	L3-L4 | 1 L4-L5 | 17 L5-S1 | 6 L2-L3-L4 | 1 L3-L4-L5 | 5	≤5° segmental motion on lateral dynamic radiographs and no translucency around the cages	66.7% | 6 months 75.0% | 12 months	-	30 (70.0)	7 (low)
Makino T, 2021b	6, 12	Porous titanium alloy + autologous bone graft + pedicle screws	L3-L4 | 2 L4-L5 | 16 L5-S1 | 4 L3-L4-L5 | 3 L4-L5-S1 | 4	≤5° segmental motion on lateral dynamic radiographs and no translucency around the cages	72.2% | 6 months 86.1% | 12 months	-	29 (69.0)	7 (low)
Schnake KJ, 2021a	12	Titanium-coated PEEK cages + autologous bone graft + pedicle screws	L3-L4 | 1 L4-L5 | 11 L5-S1 | 15	≤5° segmental motion on lateral dynamic radiographs, the presence of bridging bone, and no radiolucent line around the cages	100.0% | 12 months	0.0%	27 (50.6)	6 (low)
Schnake KJ, 2021b	12	PEEK cages + autologous bone graft + pedicle screws	L2-L3 | 2 L3-L4 | 3 L4-L5 | 16 L5-S1 | 7	≤5° segmental motion on lateral dynamic radiographs, the presence of bridging bone, and no radiolucent line around the cages	100.0% | 12 months	0.0%	28 (52.9)	6 (low)
Lebhar J, 2020a	12	Tantalum cages + pedicle screws	Not reported	≤4° segmental motion on lateral dynamic radiographs, the presence of continuous cancellous bridges	100.0% | 12 months	-	48 (−)	3 (high)
Lee JH, 2020a	48	Titanium cages + autologous bone graft + pedicle screws	Not reported	≤2° segmental motion and ≤2 mm dislocation on lateral dynamic radiographs, and the presence of bridging bone	87.5% | 48 months	6.7%	30 (61.1)	6 (low)
Lee JH, 2020b	48	BGS-7 + pedicle screws	Not reported	≤2° segmental motion and ≤2 mm dislocation on lateral dynamic radiographs, and the presence of bridging bone	100.0% | 48 months	6.3%	32 (61.5)	6 (low)
Konomi T, 2020a	12, 24	Titanium cages + autologous bone graft + pedicle screws	L2-L3 | 1∗∗∗ L3-L4 | 10∗∗∗ L4-L5 | 53∗∗∗ L5-S1 | 24∗∗∗	≤3° segmental motion on lateral dynamic radiographs, without the presence of a visible gap between the endplate and cages or radiolucency around the pedicle screws	75.6% | 12 months 94.6% | 24 months	-	37 (66.0)	6 (low)
Konomi T, 2020b	12, 24	Titanium cages + autologous bone graft + cortical bone trajectory screws	L2-L3 | 1∗∗∗ L3-L4 | 10∗∗∗ L4-L5 | 53∗∗∗ L5-S1 | 24∗∗∗	≤3° segmental motion on lateral dynamic radiographs, without the presence of a visible gap between the endplate and cages or radiolucency around the pedicle screws	56.7% | 12 months 80.0% | 24 months	-	30 (66.0)	6 (low)
Lee GW, 2018a	6, 12	PEEK cages + autologous bone graft and demineralized bone matrix + pedicle screws	L4-L5 | 31	≤2 mm difference in interspinous distance on lateral dynamic radiographs and the presence of definitely continuous fusion mass inside or outside the cage	75.9% | 6 months 83.9% | 12 months	22.6%	31 (65.6)	7 (low)
Lee GW, 2018b	6, 12	PEEK cages + autologous bone graft and demineralized bone matrix + pedicle screws	L4-L5 | 114	≤2 mm difference in interspinous distance on lateral dynamic radiographs, and the presence of a definitely continuous fusion mass inside or outside the cage	79.2% | 6 months 92.1% | 12 months	20.2%	114 (66.9)	7 (low)
Lin B, 2016a	12, 24	PEEK cages + pedicle screws	L3-L4 | 7 L4-L5 | 19 L5-L6 | 9	≤4° segmental motion on lateral dynamic radiographs, the presence of bridging bone without lucency, and the absence of translation	94.1% | 12 months 100.0% | 24 months	0.0%	35 (46.1)	7 (low)
Lin B, 2016b	12, 24	Autologous cages from the lumbar spinous process and laminae + pedicle screws	L3-L4 | 7 L4-L5 | 17 L5-L6 | 10	≤4° segmental motion on lateral dynamic radiographs, the presence of bridging bone without lucency, and the absence of translation	97.1% | 12 months 100.0% | 24 months	0.0%	34 (46.8)	7 (low)
Lee JH, 2016a	6, 12	Titanium cages + autologous bone graft + pedicle screws	Not reported	≤4° segmental motion and ≤3 mm translation on lateral dynamic radiographs, and the presence of bridging bone	91.4% | 6 months 91.4% | 12 months	5.7%	35 (61.1)	7 (low)
Lee JH, 2016b	6, 12	BGS-7 + pedicle screws	Not reported	≤4° segmental motion and ≤3 mm translation on lateral dynamic radiographs, and the presence of bridging bone	89.7% | 6 months 89.7% | 12 months	10.3%	39 (61.5)	7 (low)
Han SH, 2015a	12	PEEK cages + autologous bone graft + pedicle screws	L4-L5 | 59	≤5° segmental motion and ≤2 mm translation on lateral dynamic radiographs and Brantigan-Steffee classification D or E	89.8% | 12 months	9.6%	59 (61.6)	5 (high)
Han SH, 2015b	12	PEEK cages + autologous bone graft + pedicle screws	L5-S1 | 14	≤5° segmental motion and ≤2 mm translation on lateral dynamic radiographs and Brantigan-Steffee classification D or E	42.9% | 12 months	2.7%	14 (62.6)	5 (high)
Lee GW, 2015a	6, 12	PEEK cages + autologous bone graft + pedicle screws	L4-L5 | 19 L5-S1 | 20	≤2° segmental motion on lateral dynamic radiographs and the presence of a continuous fusion mass either inside or outside the cage	64.1% | 6 months 87.2% | 12 months	38.5%	39 (51.9)	7 (low)
Lee GW, 2015b	6, 12	PEEK cages + autologous bone graft + cortical bone trajectory screws	L4-L5 | 18 L5-S1 | 20	≤2° segmental motion on lateral dynamic radiographs and the presence of a continuous fusion mass either inside or outside the cage	68.4% | 6 months 89.5% | 12 months	42.1%	38 (51.3)	7 (low)
Lee SM, 2015a	12, 24	PEEK cages + autologous bone graft and demineralized bone matrix + pedicle screws	L4-L5 | 20 L5-S1 | 17	≤2° segmental motion on lateral dynamic radiographs and the definite presence of a continuous fusion mass with no lucency either inside or outside the cage	81.1% | 12 months 91.9% | 24 months	29.7%	37 (53.4)	6 (low)
Lee SM, 2015b	12, 24	PEEK cages + autologous bone graft and demineralized bone matrix + pedicle screws	L4-L5 | 111 L5-S1 | 108	≤2° segmental motion on lateral dynamic radiographs and the definite presence of a continuous fusion mass with no lucency either inside or outside the cage	83.1% | 12 months 91.8% | 24 months	32.9%	219 (54.9)	6 (low)
Lee N, 2017a	12, 24	PEEK cages + autologous bone graft + pedicle screws	Not reported	≤4° segmental motion on lateral dynamic radiographs, the presence of trabecular continuity of grafted bone material without any visual gap	93.8% | 12 months 92.8% | 24 months	-	30 (56.5)	5 (high)
Kim KT, 2006a	6, 12	Titanium cages + autologous bone graft + pedicle screws	Not reported	≤5° segmental motion on lateral dynamic radiographs, the presence of bridging bone and the absence of radiolucency around the cages and cage migration	78.0% | 6 months 91.0% | 12 months	22.8%	57 (55.2)	7 (low)
Ham DW, 2024a	12	Titanium cages + autologous bone graft + pedicle screws	L3-L4 | 4 L4-L5 | 22 L5-S1 | 6	≤2° segmental motion on lateral dynamic radiographs	93.8% | 12 months	3.1%	32 (71.3)	7 (low)
Ham DW, 2024b	12	Titanium cages (non-window) + pedicle screws	L3-L4 | 4 L4-L5 | 20 L5-S1 | 5	≤2° segmental motion on lateral dynamic radiographs	96.6% | 12 months	0.0%	29 (70.4)	7 (low)

**Footnotes.** In case an article presented two or more treatment arms, these were treated as separate case series, as the focus of this review was on evaluating fusion rates within each treatment arm, as opposed to comparing the different surgical interventions. Titanium-coated PEEK cages and titanium alloy cages were both analyzed as titanium cages. In addition, carbon-fiber reinforced PEEK cages were analyzed as PEEK cages. Based on an adjusted version of the Dutch Cochrane Centre checklist for cohort studies, all included articles were scored based on three domains: selection bias, outcome bias, and follow-up bias. Three points could be awarded per domain, contributing to a maximum total score of 9 points. A distinction was made between studies with 6 points or more, which were classified as ‘‘low risk of bias'’ and studies with 5 points or less, which were classified as ‘‘high risk of bias'’. ∗Mean age only reported for full cohort.∗∗Fusion level and number of fusions only reported for full cohort. ∗∗∗ Fusion levels and number of fusions are only reported for the full cohort, including 11 transforaminal lumbar interbody fusions. PEEK = polyetheretherketone; BGS-7 = bioactive glass ceramic spacer.

Cage materials consisted of polyetheretherketone (PEEK), titanium, titanium-coated PEEK, carbon-fiber-reinforced PEEK, bioactive glass ceramic spacer, titanium alloy, tantalum, and autologous bone, whereas graft substrates consisted of local bone grafts, with or without demineralized bone matrix. Titanium-coated PEEK cages and titanium alloy cages were both analyzed as titanium cages. In addition, carbon-fiber reinforced PEEK cages were analyzed as PEEK cages (Table 2).

**Table 2:** Overview of cage materials and graft substrates. Titanium-coated PEEK cages and titanium alloy cages were both analyzed as titanium cages. In addition, carbon-fiber reinforced PEEK cages were analyzed as PEEK cages.

Cage material and graft substrate	Number of patients
PEEK	681
Titanium	313
Titanium-coated PEEK	125
Carbon-fiber-reinforced PEEK	89
Bioactive glass ceramic spacer	71
Titanium alloy	29
Tantalum	48
Autologous bone	34

No graft due to solid cages	134
Local bone graft	703
Local hone graft + demineralized bone matrix	401
No additional grafts	48
Not reported	104

### 3.2 Methods of measuring bony fusion

All studies used dynamic X-ray to quantify bony fusion, while none provided a quantitative definition of bony fusion on computed tomography (CT). The difference in segmental motion (Cobb angle) between flexion/extension radiographs of the operated segment was used in thirteen studies; in three studies, a combination of the difference in translation and segmental motion upon flexion/extension was used; and in one study, the difference in interspinous distance upon flexion/extension was used for the assessment of fusion. Different cut-off points were used for these quantitative descriptive criteria, ranging from ≤5° to ≤2° in segmental motion and from ≤3 mm to ≤2 mm in translation. An overview of the used cut-off values on dynamic X-ray is provided in Table 3.

**Table 3:** Overview of cut-off values used on lateral dynamic radiographs to assess bony fusion. Some studies used multiple descriptive criteria.

Cut-off levels	Studies (n)
Segmental motion ≤5°	5
Segmental motion ≤4°	4
Segmental motion ≤3°	3
Segmental motion ≤2°	4
Translation ≤3 mm	1
Translation ≤2 mm	2
Interspinous distance ≤2 mm	1

### 3.3 Risk of bias assessment

Four studies were classified as having high risk of bias (five points or less) (Yanai et al., 2020; Lebhar et al., 2020; Han et al., 2015; Lee et al., 2017), while 13 studies were categorized as having low risk of bias (six points or more) (Yamagishi et al., 2024; Ham et al., 2023, 2024; Makino et al., 2021; Schnake et al., 2021; Lee et al., 2015, 2016, 2018, 2020; Konomi et al., 2020; Lin et al., 2016; Lee and Lee, 2015; Kim et al., 2006). Small-study effect analyses were presented in the next paragraphs.

### 3.4 Fusion rates

At 6, 12 and 24 months, a comparison was made between all case series using a cut-off value of ≤3° for the difference in segmental motion compared to the remaining case series using >3°. In addition, a comparison was made between all case series using a cut-off value of ≤4° compared to the remaining case series using >4°. Sensitivity analyses at 48 months and for translation and interspinous distance were not possible due to insufficient data.

### 3.5 Fusion rates at six months

Five studies, comprising nine case series and including 412 patients, reported fusion rates at six months postoperatively (Makino et al., 2021; Lee et al., 2015, 2016, 2018; Kim et al., 2006). The overall fusion rate was determined to be 76.0% (95% CI: 69.8-81.2%). Moderate heterogeneity was observed in the analysis (I^2^: 43.2%; p-heterogeneity: 0.08) (Table 4). All five studies were of high quality.

**Table 4:** Overview of pooled fusion rates at different timepoints and for different cage materials.

Timepoint	Cage material	Case series (n)	Fusion rate	95%CI	I^2^	p-heterogeneity
**6 months**	BGS-7	1	89.7%	72.9-96.6%	NA	NA
	PEEK	4	73.1%	63.9-80.6%	28.4%	0.24
	Titanium	4	76.4%	66.5-84.1%	47.4%	0.13
	Overall	9	76.0%	69.8-81.2%	43.2%	0.08

**12 months**	BGS-7	1	89.7%	62.9-97.8%	NA	NA
	Bone graft	1	97.1%	75.7-99.7%	NA	NA
	PEEK	12	85.7%	79.0-90.5%	63.7%	0.00
	Titanium	11	85.0%	77.4-90.4%	75.0%	0.00
	Tantalum	1	99.0%	81.9-100.0%	NA	NA
	Overall	26	86.4%	82.0-89.8%	70.4%	0.00

**24 months**	Bone graft	1	98.6%	80.5-99.9%	NA	NA
	PEEK	6	90.5%	86.6-93.3%	0.0%	0.42
	Titanium	4	89.0%	82.3-93.3%	29.4%	0.24
	Overall	11	90.2%	87.1-92.6%	13.7%	0.31

**48 months**	BGS-7	1	98.5%	79.9-99.9%	NA	NA
	Titanium	1	86.7%	69.4-94.9%	NA	NA
	Overall	2	93.4%	62.6-99.2%	56.3%	0.13

Of the seven case series (n = 267) that used the Cobb angle for the evaluation of fusion at six months, three used a cut-off of ≤5°, two used ≤4°, and two used ≤2°. A cut-off value of ≤3 (two case series (n = 77)) resulted in a fusion rate of 66.2% (95% CI: 51.4-78.4%) (I^2^: 0.0%; p-heterogeneity: 0.69) compared to a fusion rate of 79.0% (95% CI: 70.5-85.5%) (I^2^: 55.5%; p-heterogeneity: 0.06) in studies with a cut-off value of >3° (five case series (n = 190)). A cut-off value of ≤4° (four case series (n = 151)) yielded a fusion rate of 78.0% (95% CI: 66.1-86.5%) (I^2^: 74.6%; p-heterogeneity: 0.01) in contrast to studies that used a cut-off value of >4° (three case series (n = 116)) resulting in 72.6% fusion rate (95% CI: 58.4-83.3%) (I^2^: 0.0%; p-heterogeneity: 0.57) (Table 5).

**Table 5:** Overview of pooled fusion rates at different timepoints and for different cut-off values for the difference in segmental motion upon flexion/extension assessed by dynamic X-ray.

Timepoint	Cut-off value	Number of patients | case series	Fusion rate	95% CI fusion rate	I^2^	p-heterogeneity
**6 months**	≤3°	77 | 2	66.2%	51.4-78.4%	0.0%	0.69
	>3°	190 | 5	79.0%	70.5-85.5%	55.5%	0.06

	≤4°	151 | 4	78.0%	66.1-86.5%	74.6%	0.01
	>4°	116 | 3	72.6%	58.4-83.3%	0.0%	0.57

**12 months**	≤3°	561 | 9	83.3%	74.6-89.5%	75.8%	0.00
	>3°	553 | 15	88.1%	82.2-92.2%	63.9%	0.00

	≤4°	782 | 15	87.7%	81.8-91.8%	73.6%	0.00
	>4°	332 | 9	83.8%	74.1-90.4%	67.1%	0.00

**24 months**	≤3°	392 | 6	89.6%	84.8-93.0%	28.0%	0.23
	>3°	256 | 5	91.3%	85.7-94.8%	12.0%	0.34

	≤4°	491 | 9	90.7%	86.7-93.6%	29.0%	0.19
	>4°	157 | 2	89.2%	81.6-93.9%	0.0%	0.85

### 3.6 Fusion rates at 12 months

**Fig. 2:**
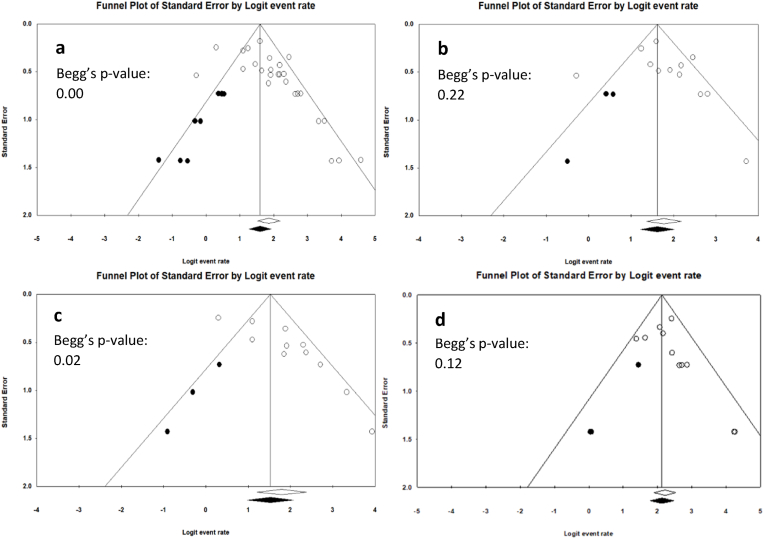
Funnel plots of the standard error against the logit event rate generated for **a)** all cages at the 12-month interval (26 included case series (I^2^: 79.4%; p-heterogeneity: 0.00%) and eight imputed potentially missing records), **b)** PEEK cages at the 12-month interval (12 included case series (I^2^: 64.0%; p-heterogeneity: 0.00) and three imputed potentially missing records), **c)** titanium cages at the 12-month interval (11 included case series (I^2^: 75.0%; p-heterogeneity: 0.00) and three imputed potentially missing records) and **d)** all cages at the 24-month interval (11 included case series (I^2^: 13.7%; p-heterogeneity: 0.31) and three imputed potentially missing records). The vertical line represents the logit of the overall pooled fusion rate, and the two oblique lines depict the 95% confidence interval for a specific standard error. The Begg's test was performed to check for asymmetry in the funnel plot, and the p-value is displayed in each graph. The trim-and-fill method was conducted to further diagnose a potential publication bias. The clear diamond indicates the pooled fusion rate of all included studies as represented by the clear dots, while the black diamond indicates the adjusted fusion rate after imputing potentially missing records showing a lower fusion rate, as represented by the black dots.

Fifteen studies, including 26 case series and 1259 patients, reported fusion rates at 12 months postoperatively (Yamagishi et al., 2024; Ham et al., 2023, 2024; Makino et al., 2021; Schnake et al., 2021; Lebhar et al., 2020; Konomi et al., 2020; Lee et al., 2015, 2016, 2017, 2018; Lin et al., 2016; Han et al., 2015; Lee and Lee, 2015; Kim et al., 2006). The overall fusion rate was calculated to be 86.4% (95% CI: 82.0-89.8%). The analysis showed substantial heterogeneity (I^2^: 70.4%; p-heterogeneity: 0.00) (Table 4). Excluding the three studies of low quality did not result in a significant change in the overall fusion rate, as this was 86.7% (95% CI: 81.9-90.4%) (I^2^: 67.2%; p-heterogeneity: 0.00) (Lebhar et al., 2020; Han et al., 2015; Lee et al., 2017). Asymmetry in the funnel plot and the Begg's test were indicative of small study effects (the phenomenon that smaller studies often show larger treatment effects than large ones, at least in part, due to publication bias) (p = 0.00) (Fig. 2a). The trim-and-fill method was used to impute the eight potentially missing articles due to publication bias, resulting in an adjusted fusion rate of 83.1% (95% CI: 78.1-87.2%). The effect of publication bias was further investigated for titanium and PEEK separately, as these are the only cage materials with ten or more case series included at 12 months. There was no evidence of small study effects for PEEK based on Begg's test (p = 0.22) (Fig. 2b). Nevertheless, because of visual assessment of asymmetry, the trim-and-fill method was used to correct for potential publication bias. Three potentially missing studies were imputed, causing a decrease in fusion rate from 85.5% (95% CI: 79.7-89.8%) to 83.4% (95% CI: 77.2-88.2%) for PEEK cages. For titanium, small study effects were demonstrated, derived from the asymmetry in the funnel plot and the Begg's test (p = 0.02) (Fig. 2c). The trim-and-fill method resulted in the imputation of three potentially missing studies and the decrease in fusion rate from to 85.7% (95% CI: 77.0-91.5%) to 82.1% (95% CI: 72.7-88.8%) for titanium cages.

Twenty-four case series (n = 1114) quantified fusion using Cobb angles. Nine case series that reported fusion rates at 12 months used a cut-off for the difference in Cobb angle of ≤5°, while six case series used a cut-off value of ≤4°, three case series used a cut-off of ≤3°, and six case series used a cut-off of ≤2°. Studies with a cut-off value of ≤3° (nine case series (n = 561)) yielded a fusion rate of 83.3% (95% CI: 74.6-89.5%) (I^2^: 75.8%; p-heterogeneity: 0.00) and with a cut-off value of >3° (15 case series (n = 553)) 88.1% fusion was demonstrated (95% CI: 82.2-92.2%) (I^2^: 63.9%; p-heterogeneity: 0.00). Studies with a cut-off value of ≤4° (15 case series (n = 782)) yielded a 87.7% fusion rate (95% CI: 81.8-91.8%) (I^2^: 73.6%; p-heterogeneity: 0.00) versus 83.8% fusion (95% CI: 74.1-90.4%) (I^2^: 67.1%; p-heterogeneity: 0.00) in studies with a cut-off value of >4° (nine case series (n = 332)) (Table 5).

### 3.7 Fusion rates at 24 months

Six studies, comprising 11 case series and including 648 patients, reported fusion rates at 24 months postoperatively (Yamagishi et al., 2024; Yanai et al., 2020; Konomi et al., 2020; Lin et al., 2016; Lee and Lee, 2015; Lee et al., 2017). The overall fusion rate was determined to be 90.2% (95% CI: 87.1-92.6%). Low heterogeneity was observed in the analysis (I^2^: 13.7%; p-heterogeneity: 0.31) (Table 4). Excluding the two studies of low quality did not significantly change the overall fusion rate, as this was 90.5% (95% CI: 86.7-93.3%) (I^2^: 23.0%; p-heterogeneity: 0.25) (Yanai et al., 2020; Lee et al., 2017). Despite Begg's test being nonsignificant (p = 0.12) for small study effects, the trim-and-fill method was used to correct for potential publication bias, resulting in the imputation of three potentially missing studies and an adjusted fusion rate of 89.4% (95% CI: 85.7-92.2%) (Fig. 2d).

All 11 case series quantified fusion using Cobb angles at 24 months. Two case series used a cut-off value of ≤5°, while three case series used a cut-off value of ≤4°, four case series used a cut-off value of ≤3° and two case series used a cut-off of ≤2°.

Studies using a cut-off value of ≤3° (six case series (n = 392)) yielded a fusion rate of 89.6% (95% CI: 84.8-93.0%) (I^2^: 28.0%; p-heterogeneity: 0.23) and studies that used a cut-off value of >3° (five case series (n = 256)) demonstrated a fusion rate of 91.3% (95% CI: 85.7-94.8%) (I^2^: 12.0%; p-heterogeneity: 0.34). Studies using a cut-off value of ≤4° (nine case series (n = 491)) had a fusion rate of 90.7% (95% CI: 86.7-93.6%) (I^2^: 29.0%; p-heterogeneity: 0.19) and studies with >4° (two case series (n = 157)) as cut-off value showed a fusion rate of 89.2% (95% CI: 81.6-93.9%) (I^2^: 0.0%; p-heterogeneity: 0.85) (Table 5).

### 3.8 Fusion rates at 48 months

One study of high quality, consisting of two case series and including 62 patients, reported fusion rates at 48 months postoperatively (Lee et al., 2020). The overall fusion rate was calculated to be 93.4% (95% CI: 62.6-99.2%). There was substantial heterogeneity in the analysis (I^2^: 56.3%; p-heterogeneity: 0.13) (Table 4).

### 3.9 Subgroup analysis of pooled fusion rates by cage type at each follow-up time

Pooled fusion rates were comparable for PEEK and titanium at 6, 12, and 24 months, as can be seen in Table 3. There was insufficient data to evaluate this relationship at 48 months. Furthermore, bone graft, bioactive glass ceramic spacer (BGS-7), and tantalum demonstrated the highest fusion rates at any given timepoint.

### 3.10 Meta-regression of pooled fusion rate by percentage smokers

There was not enough data on smoking to perform meta-regression at 6, 24, and 48 months postoperatively. Meta-regression was performed to evaluate the effect of smoking on the fusion rates at 12 months postoperatively. Seventeen case series, including 175 smokers and 669 non-smokers, were evaluated at the 12-month timepoint, and the pooled incidence of fusion rate did not seem to vary as the percent smokers varied: slope: −0.0152 (95% CI: −0.0428-0.0124; p = 0.28).

### 3.11 One study removal analysis

Two outlying case series were identified with regard to the fusion rate at 12 months, demonstrating fusion rates of 56.7% and 42.9% using titanium and PEEK cages (Table 1) (Konomi et al., 2020; Han et al., 2015). One study removal analysis was performed to evaluate the effect of the removal of each study at a time, including the outlying case series for both cage materials separately. Removal of the outlier changed the pooled fusion rate for titanium cages from 85.7% (95% CI: 77.0-91.5%) (I^2^: 75.0%; p-heterogeneity: 0.00) to 87.0% (95% CI: 81.0-91.3%) (I^2^: 44.3%; p-heterogeneity: 0.06). Removing the outlying case series for PEEK cages, changed the pooled fusion rate from 85.5% (95% CI: 79.7-89.8%) (I^2^: 63.7%; p-heterogeneity: 0.001) to 86.7% (95% CI: 82.5-90.0%) (I^2^: 39.8%; p-heterogeneity: 0.08).

## 4 Discussion

This systematic review and meta-analysis aimed to provide insights into fusion rates at different postoperative timepoints following PLIF surgery using quantitative fusion assessment criteria. Currently, there is no gold standard to evaluate interbody fusion following PLIF, resulting in an extensive variability in fusion assessment methods (Duits et al., 2024; Yu et al., 2025b). This review demonstrated that the most commonly used quantitative fusion criterion is the difference in segmental motion on the target level upon flexion/extension on lateral dynamic radiographs. However, within the articles using this criterion there is still variation in reported fusion rates. It remains uncertain whether the observed difference is attributable to the different cut-off values chosen. Therefore, interpretation of the effect of different cut-off values for segmental motion on fusion rates should be done with caution. To assess what cut-off value is most appropriate to assess fusion, the heterogeneity was evaluated at every timepoint. At six months, the degree of heterogeneity varied across the different analyses and cut-off value subgroups. This variation can partly be explained by the limited number of included case series in some subgroups, with certain analyses being based on only two case series. This reduces the reliability of heterogeneity estimates and subgroup comparisons. Therefore, it is more appropriate to base conclusions regarding cut-off values primarily on the 12- and 24-month outcome data. At 12 months, heterogeneity is present but does not significantly differ between the various Cobb angle cut-off definitions, whereas at 24 months, heterogeneity is consistently low across all analyses. Across the 12- and 24-month timepoints, a difference in Cobb angle cut-off of ≤3° and ≤4° both appear to be an appropriate threshold for the evaluation of fusion. These cut-off values result in fusion rates that are comparable.

Two additional quantitative criteria, translation and interspinous distance, were described. However, sensitivity analyses were not feasible because translation was reported in only three studies and interspinous distance in a single study. There is limited evidence supporting the use of differences in distance between adjacent spinous processes and translation during flexion and extension for the evaluation of fusion (Lee et al., 2018). Moreover, no established cut-off values exist for these two quantitative criteria, and considerable anatomical variability across vertebral levels and individuals further complicates reliable measurement (Waxenbaum et al., 2025).

This review demonstrated that fusion rates increase over time, from 76.0% at 6 months, to 86.4% at 12 months, 90.2% at 24 months, and 93.4% at 48 months postoperatively, which is important for preoperative patient counseling. In order to improve the reliability of these percentages, sensitivity analyses were applied and yielded similar fusion rate percentages varying from 66 to 78% after 6 months, 83 to 88% after 12 months, and 90 to 91%after 24 months, for respectively a cut-off value of ≤3° and ≤4°.

In addition to determining the proportion of patients demonstrating fusion at a given time point, it is equally important to investigate the extent to which the presence of fusion is associated with the patient's clinical condition. Unfortunately, the literature addressing this relationship is very limited.

The only included study that examined this showed no significant difference in ODI, NRS, SF-12 and EQ-5D scores between fused and non-fused patients, implying a lack of correlation between fusion status and clinical recovery (Ham et al., 2024). This study used the ≤3° criterion but included only 31 patients. This evidence is insufficient to determine whether radiographic fusion correlates with clinical outcomes and future research should further investigate this correlation. Furthermore, it is essential that studies examining the postoperative outcomes of interbody fusion include both clinical and radiological data to provide a comprehensive overview of the effectiveness of the procedure.

This literature overview aimed to additionally give an answer to the difference in fusion rates using PEEK or titanium intervertebral cages. With the current data, similar overall fusion rates were reported at all studied timepoints. It would have been preferable to perform sensitivity analyses using the ≤3° and ≤4° Cobb angle cut-off values for both types of cage material, but the number of case series was too limited.

Meta-analyses from 2021 to 2025 demonstrated superior fusion rates in patients undergoing lumbar fusion with titanium cages compared to PEEK cages, with an odds ratio of 2.12 (95% CI 1.05-4.28, p = 0.04) and 2.20 (95% CI 1.27-3.80, p = <0.01), respectively (Seaman et al., 2017; Wang et al., 2025). The number of patients analyzed in those meta-analyses was comparable to ours, but the described lumbar interbody fusion techniques demonstrated a lot of variation, making conclusions less generalizable. Alternative cage materials such as a bioactive glass ceramic spacer, tantalum, and autologous bone showed the highest fusion rates. However, very limited data exist on these alternative cage materials, with a maximum of one case series per cage type at each timepoint. Therefore, although these findings may suggest a true effect, they should be interpreted as preliminary, and further research is required to confirm them. Six case series evaluated fusion rates following PLIF surgery using polyaxial screws inserted via the ‘cortical bone trajectory’ (CBT) (Huedo-Medina et al., 2006; Ham et al., 2023; Han et al., 2015; Sakaura et al., 2016). In general, the reported fusion rates in those studies were comparable to the fusion rates in studies evaluating fusion rates following conventional PLIF, using pedicle screws. Therefore, the low fusion rate of 56.7% reported in one of these studies is unlikely to be attributable to a deviating screw trajectory (Sakaura et al., 2016). Likewise, a 2024 meta-analysis did not demonstrate a difference in fusion rate between CBT-PLIF and conventional PLIF (Zheng et al., 2024).

This meta-analysis failed to demonstrate a significant effect of smoking on fusion rate. However, data on smoking were very limited, with only 19 of 30 case series reporting the percentage of smokers. It was often unclear whether patients had a positive smoking history or were active smokers, and data on the average number of cigarettes a day were missing for all studies. Despite our negative result, it is well-established that smoking negatively affects fusion rate following interbody fusion (Li et al., 2021; Glassman et al., 2000; Nunna et al., 2022).

## 5 Strengths and limitations

This meta-analysis analyzed seventeen studies, allowing for a detailed temporal assessment of fusion development following PLIF. In addition, sensitivity-, stratified- and subgroup analyses were performed to evaluate the effect of cut-off values, study quality, cage material and outlier removal, enhancing the reliability of the findings. However, substantial heterogeneity was observed at each timepoint, due to variability in fusion criteria, implant characteristics and patient populations. Although the heterogeneity was substantial, the direction and temporal pattern of fusion development remained consistent across analyses. Nevertheless, limited data exist on alternative cage materials, long-term outcomes, quantitative criteria other than the difference in segmental motion, and smoking, limiting the ability to perform fully adjusted analyses. This meta-analysis focused exclusively on quantitative criteria, although most studies assessed fusion using both quantitative and qualitative measures. Due to the considerable variability in fusion definitions, we were unable to evaluate the influence of different qualitative criteria. Consequently, some patients may have been classified as “not fused” despite meeting the criteria for difference in degrees of segmental motion but not fulfilling the remaining qualitative fusion criteria, which may have influenced the results.

## 6 Conclusions

The wide variety in fusion criteria and definitions observed in this meta-analysis is in line with previously published meta-analyses which reported more than 250 unique combinations of fusion criteria to evaluate interbody fusion following a non-specific fusion procedure (Duits et al., 2024). Fusion rates described in literature reach 90% two years after PLIF surgery, and do not increase significantly during the following two years. This meta-analysis showed that a cut-off value for the difference in Cobb angle of ≤3° and ≤4° are both suitable to evaluate fusion at different post-operative timepoints and are most reliable at 24 months. Future research should focus on the correlation of fusion with clinical parameters.

## Authors’ contributions

CLAVL and LWFH formulated the research question. CLAVL, RAM and LWFH contributed to the development of the study design. CLAVL and LWFH screened abstracts, performed full-text screening and risk-of-bias assessment. LWFH extracted the data. RAM and LWFH performed the data analysis. CLAVL and LWFH interpreted the results of the data analysis. LWFH prepared the first draft of the protocol paper, with contributions and editing from CLAVL and RAM. All authors have read and approved the final manuscript.

## Funding sources

This research did not receive any specific grant from funding agencies in the public, commercial, or not-for-profit sectors.

## Declaration of competing interests

CLAVL: Thromboembolic complications in Neurosurgery (Covidien), Fundamentals of radiculopathy (YM Fund/Achmea health insurance and Eurospine), 5yr FU disc prosthesis in cervical spine surgery (CSRS E). Smoking in spondylodesis surgery (Achmea health insurance). Board of CSRS Europe, Netherlands Neurosurgical Society (NVvN) and Eurospine, Advisory Board for Rijndam Rehabilitation centre, faculty for EANS, CSRS, Eurospine spine courses. RAM and LWFH declare that they have no competing interests to disclose.

## Figures and Tables

**Figure:**
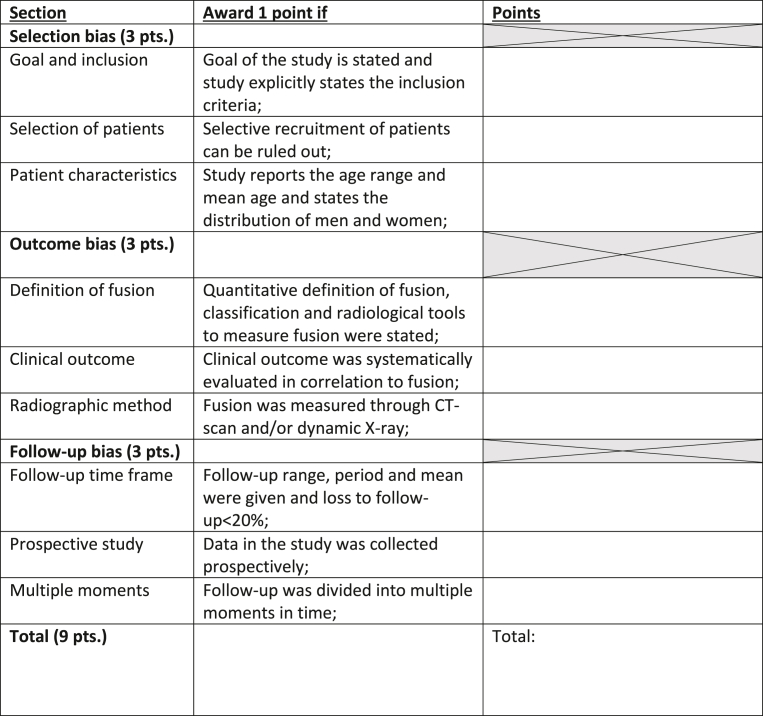


## List of abbreviations

PLIF: Posterior lumbar interbody fusion; 95% CI: 95% Confidence interval; PEEK: Polyetheretherketone; CT: Computed tomography; BGS-7: Bioactive glass ceramic spacer; CBT-PLIF: Cortical bone trajectory posterior lumbar interbody fusion.

## Appendix A Search strategy

### PubMed:

**(**(("posterior lumbar fusion"[tw] OR "posterior lumbar fusions"[tw] OR "posterior lumbar spine fusion"[tw] OR "posterior lumbar spine fusions"[tw] OR "posterior lumbosacral fusion"[tw] OR "posterior lumbar interbody fusion"[tw] OR "posterior lumbar interbody fusions"[tw] OR "posterior lumbar inter body fusion"[tw] OR "posterolateral lumbar fusion"[tw] OR "posterolateral lumbar fusions"[tw] OR "posterolateral lumbar spine fusion"[tw] OR "posterolateral lumbar spine fusions"[tw] OR "posterolateral lumbosacral fusions"[tw] OR "posterolateral lumbosacral fusion"[tw] OR "posterolateral lumbar interbody fusion"[tw] **OR** "posterior lumbar fusion"[title/abstract:∼6] OR "posterior lumbar fusions"[title/abstract:∼6] OR "posterior lumbar spine fusion"[title/abstract:∼6] OR "posterior lumbar spine fusions"[title/abstract:∼6] OR "posterior lumbosacral fusions"[title/abstract:∼6] OR "posterior lumbosacral fusion"[title/abstract:∼6] OR "posterior lumbar interbody fusion"[title/abstract:∼6] OR "posterior lumbar interbody fusions"[title/abstract:∼6] OR "posterior lumbar inter body fusion"[title/abstract:∼6] OR "posterior lumbar inter body fusions"[title/abstract:∼6] OR "posterolateral lumbar fusion"[title/abstract:∼6] OR "posterolateral lumbar fusions"[title/abstract:∼6] OR "posterolateral lumbar spine fusion"[title/abstract:∼6] OR "posterolateral lumbar spine fusions"[title/abstract:∼6] OR "posterolateral lumbosacral fusions"[title/abstract:∼6] OR "posterolateral lumbosacral fusion"[title/abstract:∼6] OR "posterolateral lumbar interbody fusion"[title/abstract:∼6] OR "posterolateral lumbar interbody fusions"[title/abstract:∼6] OR "posterolateral lumbar inter body fusion"[title/abstract:∼6] OR "posterolateral lumbar inter body fusions"[title/abstract:∼6] OR "PLIF"[tw]) AND (("Tomography, X-Ray Computed"[Mesh] AND "quantitative"[tw]) OR "QCT"[tw] OR "CAT Scan quantitative"[title/abstract:∼6] OR "CAT Scans quantitative"[title/abstract:∼6] OR "Computed Tomography quantitative"[title/abstract:∼6] OR "Computer Assisted Tomography quantitative"[title/abstract:∼6] OR "Computer Tomography quantitative"[title/abstract:∼6] OR "Computerised Axial Tomography quantitative"[title/abstract:∼6] OR "Computerised Tomography quantitative"[title/abstract:∼6] OR "Computerized Axial Tomography quantitative"[title/abstract:∼6] OR "Computerized Tomography quantitative"[title/abstract:∼6] OR "CT Scan quantitative"[title/abstract:∼6] OR "CT Scans quantitative"[title/abstract:∼6] OR "CT quantitative"[title/abstract:∼6] OR ("Radiography"[Mesh] AND "dynamic"[tw]) OR "Radiography dynamic"[title/abstract:∼6] OR "X-ray dynamic"[title/abstract:∼6] OR "Xray dynamic"[title/abstract:∼6] OR "X-rays dynamic"[title/abstract:∼6] OR "Xrays dynamic"[title/abstract:∼6] OR "roentgen dynamic"[title/abstract:∼6] OR "rontgen dynamic"[title/abstract:∼6]) NOT ("Animals"[mesh] NOT "Humans"[mesh]) NOT (("Case Reports"[ptyp] OR "case report"[tw] OR "case rep"[all fields]) NOT ("Review"[ptyp] OR "review"[tw] OR "systematic"[sb] OR "Clinical Study"[ptyp] OR "trial"[tw] OR "RCT"[tw] OR "randomized"[tw] OR "randomised"[tw] OR "random∗"[tw])) AND ("english"[la] OR "dutch"[la])) **OR** (("posterior lumbar fusion"[tw] OR "posterior lumbar fusions"[tw] OR "posterior lumbar spine fusion"[tw] OR "posterior lumbar spine fusions"[tw] OR "posterior lumbosacral fusion"[tw] OR "posterior lumbar interbody fusion"[tw] OR "posterior lumbar interbody fusions"[tw] OR "posterior lumbar inter body fusion"[tw] OR "posterolateral lumbar fusion"[tw] OR "posterolateral lumbar fusions"[tw] OR "posterolateral lumbar spine fusion"[tw] OR "posterolateral lumbar spine fusions"[tw] OR "posterolateral lumbosacral fusions"[tw] OR "posterolateral lumbosacral fusion"[tw] OR "posterolateral lumbar interbody fusion"[tw] **OR** "posterior lumbar fusion"[title/abstract:∼6] OR "posterior lumbar fusions"[title/abstract:∼6] OR "posterior lumbar spine fusion"[title/abstract:∼6] OR "posterior lumbar spine fusions"[title/abstract:∼6] OR "posterior lumbosacral fusions"[title/abstract:∼6] OR "posterior lumbosacral fusion"[title/abstract:∼6] OR "posterior lumbar interbody fusion"[title/abstract:∼6] OR "posterior lumbar interbody fusions"[title/abstract:∼6] OR "posterior lumbar inter body fusion"[title/abstract:∼6] OR "posterior lumbar inter body fusions"[title/abstract:∼6] OR "posterolateral lumbar fusion"[title/abstract:∼6] OR "posterolateral lumbar fusions"[title/abstract:∼6] OR "posterolateral lumbar spine fusion"[title/abstract:∼6] OR "posterolateral lumbar spine fusions"[title/abstract:∼6] OR "posterolateral lumbosacral fusions"[title/abstract:∼6] OR "posterolateral lumbosacral fusion"[title/abstract:∼6] OR "posterolateral lumbar interbody fusion"[title/abstract:∼6] OR "posterolateral lumbar interbody fusions"[title/abstract:∼6] OR "posterolateral lumbar inter body fusion"[title/abstract:∼6] OR "posterolateral lumbar inter body fusions"[title/abstract:∼6] OR "PLIF"[tw]) AND ("Tomography, X-Ray Computed"[majr] OR "CAT Scan"[ti] OR "CAT Scans"[ti] OR "Computed Tomogra∗"[ti] OR "Computed Tomography"[ti] OR "Computer Assisted Tomogra∗"[ti] OR "Computer Assisted Tomography"[ti] OR "Computer Tomogra∗"[ti] OR "Computer Tomography"[ti] OR "Computerised Axial Tomogra∗"[ti] OR "Computerised Axial Tomography"[ti] OR "Computerised Tomogra∗"[ti] OR "Computerised Tomography"[ti] OR "Computerized Axial Tomogra∗"[ti] OR "Computerized Axial Tomography"[ti] OR "Computerized Tomogra∗"[ti] OR "Computerized Tomography"[ti] OR "CT Scan"[ti] OR "CT Scans"[ti] OR "CT"[ti] OR "Radiography"[majr] OR "Radiography"[ti] OR "Radiogra∗"[ti] OR "X-ray"[ti] OR "Xray"[ti] OR "X-rays"[ti] OR "Xrays"[ti] OR "roentgen∗"[ti] OR "rontgen∗"[ti] OR "radiologic assessment"[ti] OR "radiologic assessments"[ti] OR "radiological assessment"[ti] OR "radiological assessments"[ti]) NOT ("Animals"[mesh] NOT "Humans"[mesh]) NOT (("Case Reports"[ptyp] OR "case report"[ti] OR "case rep"[all fields]) NOT ("Review"[ptyp] OR "review"[ti] OR "systematic"[sb] OR "Clinical Study"[ptyp] OR "trial"[ti] OR "RCT"[ti] OR "randomized"[ti] OR "randomised"[ti] OR "random∗"[ti])) AND ("english"[la] OR "dutch"[la])) **OR** (("posterior lumbar fusion"[ti] OR "posterior lumbar fusions"[ti] OR "posterior lumbar spine fusion"[ti] OR "posterior lumbar spine fusions"[ti:∼0] OR "posterior lumbosacral fusion"[ti] OR "posterior lumbar interbody fusion"[ti] OR "posterior lumbar interbody fusions"[ti] OR "posterior lumbar inter body fusion"[ti] OR "posterolateral lumbar fusion"[ti] OR "posterolateral lumbar fusions"[ti] OR "posterolateral lumbar spine fusion"[ti] OR "posterolateral lumbar spine fusions"[ti] OR "posterolateral lumbosacral fusions"[ti:∼0] OR "posterolateral lumbosacral fusion"[ti] OR "posterolateral lumbar interbody fusion"[ti] **OR** "posterior lumbar fusion"[title:∼6] OR "posterior lumbar fusions"[title:∼6] OR "posterior lumbar spine fusion"[title:∼6] OR "posterior lumbar spine fusions"[title:∼6] OR "posterior lumbosacral fusions"[title:∼6] OR "posterior lumbosacral fusion"[title:∼6] OR "posterior lumbar interbody fusion"[title:∼6] OR "posterior lumbar interbody fusions"[title:∼6] OR "posterior lumbar inter body fusion"[title:∼6] OR "posterior lumbar inter body fusions"[title:∼6] OR "posterolateral lumbar fusion"[title:∼6] OR "posterolateral lumbar fusions"[title:∼6] OR "posterolateral lumbar spine fusion"[title:∼6] OR "posterolateral lumbar spine fusions"[title:∼6] OR "posterolateral lumbosacral fusions"[title:∼6] OR "posterolateral lumbosacral fusion"[title:∼6] OR "posterolateral lumbar interbody fusion"[title:∼6] OR "posterolateral lumbar interbody fusions"[title:∼6] OR "posterolateral lumbar inter body fusion"[title:∼6] OR "posterolateral lumbar inter body fusions"[title:∼6] OR "PLIF"[ti]) AND ("Tomography, X-Ray Computed"[Mesh] OR "CAT Scan"[tw] OR "CAT Scans"[tw] OR "Computed Tomogra∗"[tw] OR "Computed Tomography"[tw] OR "Computer Assisted Tomogra∗"[tw] OR "Computer Assisted Tomography"[tw] OR "Computer Tomogra∗"[tw] OR "Computer Tomography"[tw] OR "Computerised Axial Tomogra∗"[tw] OR "Computerised Axial Tomography"[tw] OR "Computerised Tomogra∗"[tw] OR "Computerised Tomography"[tw] OR "Computerized Axial Tomogra∗"[tw] OR "Computerized Axial Tomography"[tw] OR "Computerized Tomogra∗"[tw] OR "Computerized Tomography"[tw] OR "CT Scan"[tw] OR "CT Scans"[tw] OR "CT"[tiab]) AND ("Radiography"[Mesh] OR "Radiography"[tw] OR "Radiogra∗"[tw] OR "X-ray"[tw] OR "Xray"[tw] OR "X-rays"[tw] OR "Xrays"[tw] OR "roentgen∗"[tw] OR "rontgen∗"[tw]) NOT ("Animals"[mesh] NOT "Humans"[mesh]) NOT (("Case Reports"[ptyp] OR "case report"[ti] OR "case rep"[all fields]) NOT ("Review"[ptyp] OR "review"[ti] OR "systematic"[sb] OR "Clinical Study"[ptyp] OR "trial"[ti] OR "RCT"[ti] OR "randomized"[ti] OR "randomised"[ti] OR "random∗"[ti])) AND ("english"[la] OR "dutch"[la]))**)**

### MEDLINE via OVID:

**(**(("posterior lumbar fusion".mp OR "posterior lumbar fusions".mp OR "posterior lumbar spine fusion".mp OR "posterior lumbar spine fusions".mp OR "posterior lumbosacral fusion".mp OR "posterior lumbar interbody fusion".mp OR "posterior lumbar interbody fusions".mp OR "posterior lumbar inter body fusion".mp OR "posterolateral lumbar fusion".mp OR "posterolateral lumbar fusions".mp OR "posterolateral lumbar spine fusion".mp OR "posterolateral lumbar spine fusions".mp OR "posterolateral lumbosacral fusions".mp OR "posterolateral lumbosacral fusion".mp OR "posterolateral lumbar interbody fusion".mp OR "posterior lumbosacral fusions".mp OR "posterior lumbar inter body fusions".mp OR "posterolateral lumbar interbody fusions".mp OR "posterolateral lumbar inter body fusion".mp OR "posterolateral lumbar inter body fusions".mp **OR** (("posterior" ADJ6 ″lumbar" ADJ6 ″fusion") OR ("posterior" ADJ6 ″lumbar" ADJ6 ″fusions") OR ("posterior" ADJ6 ″lumbar" ADJ6 ″spine" ADJ6 ″fusion") OR ("posterior" ADJ6 ″lumbar" ADJ6 ″spine" ADJ6 ″fusions") OR ("posterior" ADJ6 ″lumbosacral" ADJ6 ″fusions") OR ("posterior" ADJ6 ″lumbosacral" ADJ6 ″fusion") OR ("posterior" ADJ6 ″lumbar" ADJ6 ″interbody" ADJ6 ″fusion") OR ("posterior" ADJ6 ″lumbar" ADJ6 ″interbody" ADJ6 ″fusions") OR ("posterior" ADJ6 ″lumbar" ADJ6 ″inter" ADJ6 ″body" ADJ6 ″fusion") OR ("posterior" ADJ6 ″lumbar" ADJ6 ″inter" ADJ6 ″body" ADJ6 ″fusions") OR ("posterolateral" ADJ6 ″lumbar" ADJ6 ″fusion") OR ("posterolateral" ADJ6 ″lumbar" ADJ6 ″fusions") OR ("posterolateral" ADJ6 ″lumbar" ADJ6 ″spine" ADJ6 ″fusion") OR ("posterolateral" ADJ6 ″lumbar" ADJ6 ″spine" ADJ6 ″fusions") OR ("posterolateral" ADJ6 ″lumbosacral" ADJ6 ″fusions") OR ("posterolateral" ADJ6 ″lumbosacral" ADJ6 ″fusion") OR ("posterolateral" ADJ6 ″lumbar" ADJ6 ″interbody" ADJ6 ″fusion") OR ("posterolateral" ADJ6 ″lumbar" ADJ6 ″interbody" ADJ6 ″fusions") OR ("posterolateral" ADJ6 ″lumbar" ADJ6 ″inter" ADJ6 ″body" ADJ6 ″fusion") OR ("posterolateral" ADJ6 ″lumbar" ADJ6 ″inter" ADJ6 ″body" ADJ6 ″fusions")).ti,ab OR "PLIF".mp) AND ((exp "Tomography, X-Ray Computed"/AND "quantitative".mp) OR "QCT".mp OR (("CAT" ADJ6 ″Scan" ADJ6 ″quantitative") OR ("CAT" ADJ6 ″Scans" ADJ6 ″quantitative") OR ("Computed" ADJ6 ″Tomography" ADJ6 ″quantitative") OR ("Computer" ADJ6 ″Assisted" ADJ6 ″Tomography" ADJ6 ″quantitative") OR ("Computer" ADJ6 ″Tomography" ADJ6 ″quantitative") OR ("Computerised" ADJ6 ″Axial" ADJ6 ″Tomography" ADJ6 ″quantitative") OR ("Computerised" ADJ6 ″Tomography" ADJ6 ″quantitative") OR ("Computerized" ADJ6 ″Axial" ADJ6 ″Tomography" ADJ6 ″quantitative") OR ("Computerized" ADJ6 ″Tomography" ADJ6 ″quantitative") OR ("CT" ADJ6 ″Scan" ADJ6 ″quantitative") OR ("CT" ADJ6 ″Scans" ADJ6 ″quantitative") OR ("CT" ADJ6 ″quantitative")).ti,ab OR (exp "Radiography"/AND "dynamic".mp) OR (("Radiography" ADJ6 ″dynamic") OR ("X-ray" ADJ6 ″dynamic") OR ("Xray" ADJ6 ″dynamic") OR ("X-rays" ADJ6 ″dynamic") OR ("Xrays" ADJ6 ″dynamic") OR ("roentgen" ADJ6 ″dynamic") OR ("rontgen" ADJ6 ″dynamic")).ti,ab) NOT (exp "Animals"/NOT exp "Humans"/) NOT (("Case Reports"/OR "case report".mp OR "case rep".af) NOT (exp "Review"/OR "review".mp OR exp "systematic review"/OR exp "Clinical Study"/OR "trial".mp OR "RCT".mp OR "randomized".mp OR "randomised".mp OR "random∗".mp)) AND ("english".la OR "dutch".la)) **OR** (("posterior lumbar fusion".mp OR "posterior lumbar fusions".mp OR "posterior lumbar spine fusion".mp OR "posterior lumbar spine fusions".mp OR "posterior lumbosacral fusion".mp OR "posterior lumbar interbody fusion".mp OR "posterior lumbar interbody fusions".mp OR "posterior lumbar inter body fusion".mp OR "posterolateral lumbar fusion".mp OR "posterolateral lumbar fusions".mp OR "posterolateral lumbar spine fusion".mp OR "posterolateral lumbar spine fusions".mp OR "posterolateral lumbosacral fusions".mp OR "posterolateral lumbosacral fusion".mp OR "posterolateral lumbar interbody fusion".mp OR "posterior lumbosacral fusions".mp OR "posterior lumbar inter body fusions".mp OR "posterolateral lumbar interbody fusions".mp OR "posterolateral lumbar inter body fusion".mp OR "posterolateral lumbar inter body fusions".mp **OR** (("posterior" ADJ6 ″lumbar" ADJ6 ″fusion") OR ("posterior" ADJ6 ″lumbar" ADJ6 ″fusions") OR ("posterior" ADJ6 ″lumbar" ADJ6 ″spine" ADJ6 ″fusion") OR ("posterior" ADJ6 ″lumbar" ADJ6 ″spine" ADJ6 ″fusions") OR ("posterior" ADJ6 ″lumbosacral" ADJ6 ″fusions") OR ("posterior" ADJ6 ″lumbosacral" ADJ6 ″fusion") OR ("posterior" ADJ6 ″lumbar" ADJ6 ″interbody" ADJ6 ″fusion") OR ("posterior" ADJ6 ″lumbar" ADJ6 ″interbody" ADJ6 ″fusions") OR ("posterior" ADJ6 ″lumbar" ADJ6 ″inter" ADJ6 ″body" ADJ6 ″fusion") OR ("posterior" ADJ6 ″lumbar" ADJ6 ″inter" ADJ6 ″body" ADJ6 ″fusions") OR ("posterolateral" ADJ6 ″lumbar" ADJ6 ″fusion") OR ("posterolateral" ADJ6 ″lumbar" ADJ6 ″fusions") OR ("posterolateral" ADJ6 ″lumbar" ADJ6 ″spine" ADJ6 ″fusion") OR ("posterolateral" ADJ6 ″lumbar" ADJ6 ″spine" ADJ6 ″fusions") OR ("posterolateral" ADJ6 ″lumbosacral" ADJ6 ″fusions") OR ("posterolateral" ADJ6 ″lumbosacral" ADJ6 ″fusion") OR ("posterolateral" ADJ6 ″lumbar" ADJ6 ″interbody" ADJ6 ″fusion") OR ("posterolateral" ADJ6 ″lumbar" ADJ6 ″interbody" ADJ6 ″fusions") OR ("posterolateral" ADJ6 ″lumbar" ADJ6 ″inter" ADJ6 ″body" ADJ6 ″fusion") OR ("posterolateral" ADJ6 ″lumbar" ADJ6 ″inter" ADJ6 ″body" ADJ6 ″fusions")).ti,ab OR "PLIF".mp) AND (exp ∗"Tomography, X-Ray Computed"/OR "CAT Scan".ti OR "CAT Scans".ti OR "Computed Tomogra∗".ti OR "Computed Tomography".ti OR "Computer Assisted Tomogra∗".ti OR "Computer Assisted Tomography".ti OR "Computer Tomogra∗".ti OR "Computer Tomography".ti OR "Computerised Axial Tomogra∗".ti OR "Computerised Axial Tomography".ti OR "Computerised Tomogra∗".ti OR "Computerised Tomography".ti OR "Computerized Axial Tomogra∗".ti OR "Computerized Axial Tomography".ti OR "Computerized Tomogra∗".ti OR "Computerized Tomography".ti OR "CT Scan".ti OR "CT Scans".ti OR "CT".ti OR exp ∗"Radiography"/OR "Radiography".ti OR "Radiogra∗".ti OR "X-ray".ti OR "Xray".ti OR "X-rays".ti OR "Xrays".ti OR "roentgen∗".ti OR "rontgen∗".ti OR "radiologic assessment".ti OR "radiologic assessments".ti OR "radiological assessment".ti OR "radiological assessments".ti) NOT (exp "Animals"/NOT exp "Humans"/) NOT (("Case Reports"/OR "case report".ti OR "case rep".af) NOT (exp "Review"/OR "review".ti OR exp "systematic review"/OR exp "Clinical Study"/OR "trial".ti OR "RCT".ti OR "randomized".ti OR "randomised".ti OR "random∗".ti)) AND ("english".la OR "dutch".la)) **OR** (("posterior lumbar fusion".ti OR "posterior lumbar fusions".ti OR "posterior lumbar spine fusion".ti OR "posterior lumbar spine fusions".ti OR "posterior lumbosacral fusion".ti OR "posterior lumbar interbody fusion".ti OR "posterior lumbar interbody fusions".ti OR "posterior lumbar inter body fusion".ti OR "posterolateral lumbar fusion".ti OR "posterolateral lumbar fusions".ti OR "posterolateral lumbar spine fusion".ti OR "posterolateral lumbar spine fusions".ti OR "posterolateral lumbosacral fusions".ti OR "posterolateral lumbosacral fusion".ti OR "posterolateral lumbar interbody fusion".ti OR "posterior lumbosacral fusions".ti OR "posterior lumbar inter body fusions".ti OR "posterolateral lumbar interbody fusions".ti OR "posterolateral lumbar inter body fusion".ti OR "posterolateral lumbar inter body fusions".ti **OR** (("posterior" ADJ6 ″lumbar" ADJ6 ″fusion") OR ("posterior" ADJ6 ″lumbar" ADJ6 ″fusions") OR ("posterior" ADJ6 ″lumbar" ADJ6 ″spine" ADJ6 ″fusion") OR ("posterior" ADJ6 ″lumbar" ADJ6 ″spine" ADJ6 ″fusions") OR ("posterior" ADJ6 ″lumbosacral" ADJ6 ″fusions") OR ("posterior" ADJ6 ″lumbosacral" ADJ6 ″fusion") OR ("posterior" ADJ6 ″lumbar" ADJ6 ″interbody" ADJ6 ″fusion") OR ("posterior" ADJ6 ″lumbar" ADJ6 ″interbody" ADJ6 ″fusions") OR ("posterior" ADJ6 ″lumbar" ADJ6 ″inter" ADJ6 ″body" ADJ6 ″fusion") OR ("posterior" ADJ6 ″lumbar" ADJ6 ″inter" ADJ6 ″body" ADJ6 ″fusions") OR ("posterolateral" ADJ6 ″lumbar" ADJ6 ″fusion") OR ("posterolateral" ADJ6 ″lumbar" ADJ6 ″fusions") OR ("posterolateral" ADJ6 ″lumbar" ADJ6 ″spine" ADJ6 ″fusion") OR ("posterolateral" ADJ6 ″lumbar" ADJ6 ″spine" ADJ6 ″fusions") OR ("posterolateral" ADJ6 ″lumbosacral" ADJ6 ″fusions") OR ("posterolateral" ADJ6 ″lumbosacral" ADJ6 ″fusion") OR ("posterolateral" ADJ6 ″lumbar" ADJ6 ″interbody" ADJ6 ″fusion") OR ("posterolateral" ADJ6 ″lumbar" ADJ6 ″interbody" ADJ6 ″fusions") OR ("posterolateral" ADJ6 ″lumbar" ADJ6 ″inter" ADJ6 ″body" ADJ6 ″fusion") OR ("posterolateral" ADJ6 ″lumbar" ADJ6 ″inter" ADJ6 ″body" ADJ6 ″fusions")).ti OR "PLIF".ti) AND (exp "Tomography, X-Ray Computed"/OR "CAT Scan".mp OR "CAT Scans".mp OR "Computed Tomogra∗".mp OR "Computed Tomography".mp OR "Computer Assisted Tomogra∗".mp OR "Computer Assisted Tomography".mp OR "Computer Tomogra∗".mp OR "Computer Tomography".mp OR "Computerised Axial Tomogra∗".mp OR "Computerised Axial Tomography".mp OR "Computerised Tomogra∗".mp OR "Computerised Tomography".mp OR "Computerized Axial Tomogra∗".mp OR "Computerized Axial Tomography".mp OR "Computerized Tomogra∗".mp OR "Computerized Tomography".mp OR "CT Scan".mp OR "CT Scans".mp OR "CT".ti,ab) AND (exp "Radiography"/OR "Radiography".mp OR "Radiogra∗".mp OR "X-ray".mp OR "Xray".mp OR "X-rays".mp OR "Xrays".mp OR "roentgen∗".mp OR "rontgen∗".mp) NOT (exp "Animals"/NOT exp "Humans"/) NOT (("Case Reports"/OR "case report".ti OR "case rep".af) NOT (exp "Review"/OR "review".ti OR exp "systematic review"/OR exp "Clinical Study"/OR "trial".ti OR "RCT".ti OR "randomized".ti OR "randomised".ti OR "random∗".ti)) AND ("english".la OR "dutch".la))**)**

### Embase:

**(**(("posterior lumbar fusion".ti,ab OR "posterior lumbar fusions".ti,ab OR "posterior lumbar spine fusion".ti,ab OR "posterior lumbar spine fusions".ti,ab OR "posterior lumbosacral fusion".ti,ab OR "posterior lumbar interbody fusion".ti,ab OR "posterior lumbar interbody fusions".ti,ab OR "posterior lumbar inter body fusion".ti,ab OR "posterolateral lumbar fusion".ti,ab OR "posterolateral lumbar fusions".ti,ab OR "posterolateral lumbar spine fusion".ti,ab OR "posterolateral lumbar spine fusions".ti,ab OR "posterolateral lumbosacral fusions".ti,ab OR "posterolateral lumbosacral fusion".ti,ab OR "posterolateral lumbar interbody fusion".ti,ab OR "posterior lumbosacral fusions".ti,ab OR "posterior lumbar inter body fusions".ti,ab OR "posterolateral lumbar interbody fusions".ti,ab OR "posterolateral lumbar inter body fusion".ti,ab OR "posterolateral lumbar inter body fusions".ti,ab **OR** (("posterior" ADJ6 ″lumbar" ADJ6 ″fusion") OR ("posterior" ADJ6 ″lumbar" ADJ6 ″fusions") OR ("posterior" ADJ6 ″lumbar" ADJ6 ″spine" ADJ6 ″fusion") OR ("posterior" ADJ6 ″lumbar" ADJ6 ″spine" ADJ6 ″fusions") OR ("posterior" ADJ6 ″lumbosacral" ADJ6 ″fusions") OR ("posterior" ADJ6 ″lumbosacral" ADJ6 ″fusion") OR ("posterior" ADJ6 ″lumbar" ADJ6 ″interbody" ADJ6 ″fusion") OR ("posterior" ADJ6 ″lumbar" ADJ6 ″interbody" ADJ6 ″fusions") OR ("posterior" ADJ6 ″lumbar" ADJ6 ″inter" ADJ6 ″body" ADJ6 ″fusion") OR ("posterior" ADJ6 ″lumbar" ADJ6 ″inter" ADJ6 ″body" ADJ6 ″fusions") OR ("posterolateral" ADJ6 ″lumbar" ADJ6 ″fusion") OR ("posterolateral" ADJ6 ″lumbar" ADJ6 ″fusions") OR ("posterolateral" ADJ6 ″lumbar" ADJ6 ″spine" ADJ6 ″fusion") OR ("posterolateral" ADJ6 ″lumbar" ADJ6 ″spine" ADJ6 ″fusions") OR ("posterolateral" ADJ6 ″lumbosacral" ADJ6 ″fusions") OR ("posterolateral" ADJ6 ″lumbosacral" ADJ6 ″fusion") OR ("posterolateral" ADJ6 ″lumbar" ADJ6 ″interbody" ADJ6 ″fusion") OR ("posterolateral" ADJ6 ″lumbar" ADJ6 ″interbody" ADJ6 ″fusions") OR ("posterolateral" ADJ6 ″lumbar" ADJ6 ″inter" ADJ6 ″body" ADJ6 ″fusion") OR ("posterolateral" ADJ6 ″lumbar" ADJ6 ″inter" ADJ6 ″body" ADJ6 ″fusions")).ti,ab OR "PLIF".ti,ab) AND ((exp ∗"Computer Assisted Tomography"/AND "quantitative".ti,ab) OR "QCT".ti,ab OR (("CAT" ADJ6 ″Scan" ADJ6 ″quantitative") OR ("CAT" ADJ6 ″Scans" ADJ6 ″quantitative") OR ("Computed" ADJ6 ″Tomography" ADJ6 ″quantitative") OR ("Computer" ADJ6 ″Assisted" ADJ6 ″Tomography" ADJ6 ″quantitative") OR ("Computer" ADJ6 ″Tomography" ADJ6 ″quantitative") OR ("Computerised" ADJ6 ″Axial" ADJ6 ″Tomography" ADJ6 ″quantitative") OR ("Computerised" ADJ6 ″Tomography" ADJ6 ″quantitative") OR ("Computerized" ADJ6 ″Axial" ADJ6 ″Tomography" ADJ6 ″quantitative") OR ("Computerized" ADJ6 ″Tomography" ADJ6 ″quantitative") OR ("CT" ADJ6 ″Scan" ADJ6 ″quantitative") OR ("CT" ADJ6 ″Scans" ADJ6 ″quantitative") OR ("CT" ADJ6 ″quantitative")).ti,ab OR (exp ∗"Radiography"/AND "dynamic".ti,ab) OR (("Radiography" ADJ6 ″dynamic") OR ("X-ray" ADJ6 ″dynamic") OR ("Xray" ADJ6 ″dynamic") OR ("X-rays" ADJ6 ″dynamic") OR ("Xrays" ADJ6 ″dynamic") OR ("roentgen" ADJ6 ″dynamic") OR ("rontgen" ADJ6 ″dynamic")).ti,ab) NOT (exp "Animals"/NOT exp "Humans"/) NOT (("Case Reports"/OR "case report".ti,ab OR (case AND (report OR reports)).jw) NOT (exp "Review"/OR "review".ti,ab OR exp "systematic review"/OR "Clinical Study"/OR exp "Clinical Trial"/OR "trial".ti,ab OR "RCT".ti,ab OR "randomized".ti,ab OR "randomised".ti,ab OR "random∗".ti,ab)) AND ("english".la OR "dutch".la)) OR (("posterior lumbar fusion".ti,ab OR "posterior lumbar fusions".ti,ab OR "posterior lumbar spine fusion".ti,ab OR "posterior lumbar spine fusions".ti,ab OR "posterior lumbosacral fusion".ti,ab OR "posterior lumbar interbody fusion".ti,ab OR "posterior lumbar interbody fusions".ti,ab OR "posterior lumbar inter body fusion".ti,ab OR "posterolateral lumbar fusion".ti,ab OR "posterolateral lumbar fusions".ti,ab OR "posterolateral lumbar spine fusion".ti,ab OR "posterolateral lumbar spine fusions".ti,ab OR "posterolateral lumbosacral fusions".ti,ab OR "posterolateral lumbosacral fusion".ti,ab OR "posterolateral lumbar interbody fusion".ti,ab OR "posterior lumbosacral fusions".ti,ab OR "posterior lumbar inter body fusions".ti,ab OR "posterolateral lumbar interbody fusions".ti,ab OR "posterolateral lumbar inter body fusion".ti,ab OR "posterolateral lumbar inter body fusions".ti,ab OR (("posterior" ADJ6 ″lumbar" ADJ6 ″fusion") OR ("posterior" ADJ6 ″lumbar" ADJ6 ″fusions") OR ("posterior" ADJ6 ″lumbar" ADJ6 ″spine" ADJ6 ″fusion") OR ("posterior" ADJ6 ″lumbar" ADJ6 ″spine" ADJ6 ″fusions") OR ("posterior" ADJ6 ″lumbosacral" ADJ6 ″fusions") OR ("posterior" ADJ6 ″lumbosacral" ADJ6 ″fusion") OR ("posterior" ADJ6 ″lumbar" ADJ6 ″interbody" ADJ6 ″fusion") OR ("posterior" ADJ6 ″lumbar" ADJ6 ″interbody" ADJ6 ″fusions") OR ("posterior" ADJ6 ″lumbar" ADJ6 ″inter" ADJ6 ″body" ADJ6 ″fusion") OR ("posterior" ADJ6 ″lumbar" ADJ6 ″inter" ADJ6 ″body" ADJ6 ″fusions") OR ("posterolateral" ADJ6 ″lumbar" ADJ6 ″fusion") OR ("posterolateral" ADJ6 ″lumbar" ADJ6 ″fusions") OR ("posterolateral" ADJ6 ″lumbar" ADJ6 ″spine" ADJ6 ″fusion") OR ("posterolateral" ADJ6 ″lumbar" ADJ6 ″spine" ADJ6 ″fusions") OR ("posterolateral" ADJ6 ″lumbosacral" ADJ6 ″fusions") OR ("posterolateral" ADJ6 ″lumbosacral" ADJ6 ″fusion") OR ("posterolateral" ADJ6 ″lumbar" ADJ6 ″interbody" ADJ6 ″fusion") OR ("posterolateral" ADJ6 ″lumbar" ADJ6 ″interbody" ADJ6 ″fusions") OR ("posterolateral" ADJ6 ″lumbar" ADJ6 ″inter" ADJ6 ″body" ADJ6 ″fusion") OR ("posterolateral" ADJ6 ″lumbar" ADJ6 ″inter" ADJ6 ″body" ADJ6 ″fusions")).ti,ab OR "PLIF".ti,ab) AND (exp ∗"Computer Assisted Tomography"/OR "CAT Scan".ti OR "CAT Scans".ti OR "Computed Tomogra∗".ti OR "Computed Tomography".ti OR "Computer Assisted Tomogra∗".ti OR "Computer Assisted Tomography".ti OR "Computer Tomogra∗".ti OR "Computer Tomography".ti OR "Computerised Axial Tomogra∗".ti OR "Computerised Axial Tomography".ti OR "Computerised Tomogra∗".ti OR "Computerised Tomography".ti OR "Computerized Axial Tomogra∗".ti OR "Computerized Axial Tomography".ti OR "Computerized Tomogra∗".ti OR "Computerized Tomography".ti OR "CT Scan".ti OR "CT Scans".ti OR "CT".ti OR exp ∗"Radiography"/OR "Radiography".ti OR "Radiogra∗".ti OR "X-ray".ti OR "Xray".ti OR "X-rays".ti OR "Xrays".ti OR "roentgen∗".ti OR "rontgen∗".ti OR "radiologic assessment".ti OR "radiologic assessments".ti OR "radiological assessment".ti OR "radiological assessments".ti) NOT (exp "Animals"/NOT exp "Humans"/) NOT (("Case Reports"/OR "case report".ti OR (case AND (report OR reports)).jw) NOT (exp "Review"/OR "review".ti OR exp "systematic review"/OR "Clinical Study"/OR exp "Clinical Trial"/OR "trial".ti OR "RCT".ti OR "randomized".ti OR "randomised".ti OR "random∗".ti)) AND ("english".la OR "dutch".la)) OR (("posterior lumbar fusion".ti OR "posterior lumbar fusions".ti OR "posterior lumbar spine fusion".ti OR "posterior lumbar spine fusions".ti OR "posterior lumbosacral fusion".ti OR "posterior lumbar interbody fusion".ti OR "posterior lumbar interbody fusions".ti OR "posterior lumbar inter body fusion".ti OR "posterolateral lumbar fusion".ti OR "posterolateral lumbar fusions".ti OR "posterolateral lumbar spine fusion".ti OR "posterolateral lumbar spine fusions".ti OR "posterolateral lumbosacral fusions".ti OR "posterolateral lumbosacral fusion".ti OR "posterolateral lumbar interbody fusion".ti OR "posterior lumbosacral fusions".ti OR "posterior lumbar inter body fusions".ti OR "posterolateral lumbar interbody fusions".ti OR "posterolateral lumbar inter body fusion".ti OR "posterolateral lumbar inter body fusions".ti OR (("posterior" ADJ6 ″lumbar" ADJ6 ″fusion") OR ("posterior" ADJ6 ″lumbar" ADJ6 ″fusions") OR ("posterior" ADJ6 ″lumbar" ADJ6 ″spine" ADJ6 ″fusion") OR ("posterior" ADJ6 ″lumbar" ADJ6 ″spine" ADJ6 ″fusions") OR ("posterior" ADJ6 ″lumbosacral" ADJ6 ″fusions") OR ("posterior" ADJ6 ″lumbosacral" ADJ6 ″fusion") OR ("posterior" ADJ6 ″lumbar" ADJ6 ″interbody" ADJ6 ″fusion") OR ("posterior" ADJ6 ″lumbar" ADJ6 ″interbody" ADJ6 ″fusions") OR ("posterior" ADJ6 ″lumbar" ADJ6 ″inter" ADJ6 ″body" ADJ6 ″fusion") OR ("posterior" ADJ6 ″lumbar" ADJ6 ″inter" ADJ6 ″body" ADJ6 ″fusions") OR ("posterolateral" ADJ6 ″lumbar" ADJ6 ″fusion") OR ("posterolateral" ADJ6 ″lumbar" ADJ6 ″fusions") OR ("posterolateral" ADJ6 ″lumbar" ADJ6 ″spine" ADJ6 ″fusion") OR ("posterolateral" ADJ6 ″lumbar" ADJ6 ″spine" ADJ6 ″fusions") OR ("posterolateral" ADJ6 ″lumbosacral" ADJ6 ″fusions") OR ("posterolateral" ADJ6 ″lumbosacral" ADJ6 ″fusion") OR ("posterolateral" ADJ6 ″lumbar" ADJ6 ″interbody" ADJ6 ″fusion") OR ("posterolateral" ADJ6 ″lumbar" ADJ6 ″interbody" ADJ6 ″fusions") OR ("posterolateral" ADJ6 ″lumbar" ADJ6 ″inter" ADJ6 ″body" ADJ6 ″fusion") OR ("posterolateral" ADJ6 ″lumbar" ADJ6 ″inter" ADJ6 ″body" ADJ6 ″fusions")).ti OR "PLIF".ti) AND (exp ∗"Computer Assisted Tomography"/OR "CAT Scan".ti,ab OR "CAT Scans".ti,ab OR "Computed Tomogra∗".ti,ab OR "Computed Tomography".ti,ab OR "Computer Assisted Tomogra∗".ti,ab OR "Computer Assisted Tomography".ti,ab OR "Computer Tomogra∗".ti,ab OR "Computer Tomography".ti,ab OR "Computerised Axial Tomogra∗".ti,ab OR "Computerised Axial Tomography".ti,ab OR "Computerised Tomogra∗".ti,ab OR "Computerised Tomography".ti,ab OR "Computerized Axial Tomogra∗".ti,ab OR "Computerized Axial Tomography".ti,ab OR "Computerized Tomogra∗".ti,ab OR "Computerized Tomography".ti,ab OR "CT Scan".ti,ab OR "CT cans".ti,ab OR "CT".ti,ab) AND (exp ∗"Radiography"/OR "Radiography".ti,ab OR "Radiogra∗".ti,ab OR "X-ray".ti,ab OR "Xray".ti,ab OR "X-rays".ti,ab OR "Xrays".ti,ab OR "roentgen∗".ti,ab OR "rontgen∗".ti,ab) NOT (exp "Animals"/NOT exp "Humans"/) NOT (("Case Reports"/OR "case report".ti OR (case AND (report OR reports)).jw) NOT (exp "Review"/OR "review".ti OR exp "systematic review"/OR "Clinical Study"/OR exp "Clinical Trial"/OR "trial".ti OR "RCT".ti OR "randomized".ti OR "randomised".ti OR "random∗".ti)) AND ("english".la OR "dutch".la))) NOT (conference review or conference abstract).pt.

### Web of Science:

**(**(TS=("posterior lumbar fusion" OR "posterior lumbar fusions" OR "posterior lumbar spine fusion" OR "posterior lumbar spine fusions" OR "posterior lumbosacral fusion" OR "posterior lumbar interbody fusion" OR "posterior lumbar interbody fusions" OR "posterior lumbar inter body fusion" OR "posterolateral lumbar fusion" OR "posterolateral lumbar fusions" OR "posterolateral lumbar spine fusion" OR "posterolateral lumbar spine fusions" OR "posterolateral lumbosacral fusions" OR "posterolateral lumbosacral fusion" OR "posterolateral lumbar interbody fusion" OR "posterior lumbosacral fusions" OR "posterior lumbar inter body fusions" OR "posterolateral lumbar interbody fusions" OR "posterolateral lumbar inter body fusion" OR "posterolateral lumbar inter body fusions" OR (("posterior" NEAR/6 ″lumbar" NEAR/6 ″fusion") OR ("posterior" NEAR/6 ″lumbar" NEAR/6 ″fusions") OR ("posterior" NEAR/6 ″lumbar" NEAR/6 ″spine" NEAR/6 ″fusion") OR ("posterior" NEAR/6 ″lumbar" NEAR/6 ″spine" NEAR/6 ″fusions") OR ("posterior" NEAR/6 ″lumbosacral" NEAR/6 ″fusions") OR ("posterior" NEAR/6 ″lumbosacral" NEAR/6 ″fusion") OR ("posterior" NEAR/6 ″lumbar" NEAR/6 ″interbody" NEAR/6 ″fusion") OR ("posterior" NEAR/6 ″lumbar" NEAR/6 ″interbody" NEAR/6 ″fusions") OR ("posterior" NEAR/6 ″lumbar" NEAR/6 ″inter" NEAR/6 ″body" NEAR/6 ″fusion") OR ("posterior" NEAR/6 ″lumbar" NEAR/6 ″inter" NEAR/6 ″body" NEAR/6 ″fusions") OR ("posterolateral" NEAR/6 ″lumbar" NEAR/6 ″fusion") OR ("posterolateral" NEAR/6 ″lumbar" NEAR/6 ″fusions") OR ("posterolateral" NEAR/6 ″lumbar" NEAR/6 ″spine" NEAR/6 ″fusion") OR ("posterolateral" NEAR/6 ″lumbar" NEAR/6 ″spine" NEAR/6 ″fusions") OR ("posterolateral" NEAR/6 ″lumbosacral" NEAR/6 ″fusions") OR ("posterolateral" NEAR/6 ″lumbosacral" NEAR/6 ″fusion") OR ("posterolateral" NEAR/6 ″lumbar" NEAR/6 ″interbody" NEAR/6 ″fusion") OR ("posterolateral" NEAR/6 ″lumbar" NEAR/6 ″interbody" NEAR/6 ″fusions") OR ("posterolateral" NEAR/6 ″lumbar" NEAR/6 ″inter" NEAR/6 ″body" NEAR/6 ″fusion") OR ("posterolateral" NEAR/6 ″lumbar" NEAR/6 ″inter" NEAR/6 ″body" NEAR/6 ″fusions")) OR "PLIF") AND TS=(("Computer Assisted Tomography" AND "quantitative") OR "QCT" OR (("CAT" NEAR/6 ″Scan" NEAR/6 ″quantitative") OR ("CAT" NEAR/6 ″Scans" NEAR/6 ″quantitative") OR ("Computed" NEAR/6 ″Tomography" NEAR/6 ″quantitative") OR ("Computer" NEAR/6 ″Assisted" NEAR/6 ″Tomography" NEAR/6 ″quantitative") OR ("Computer" NEAR/6 ″Tomography" NEAR/6 ″quantitative") OR ("Computerised" NEAR/6 ″Axial" NEAR/6 ″Tomography" NEAR/6 ″quantitative") OR ("Computerised" NEAR/6 ″Tomography" NEAR/6 ″quantitative") OR ("Computerized" NEAR/6 ″Axial" NEAR/6 ″Tomography" NEAR/6 ″quantitative") OR ("Computerized" NEAR/6 ″Tomography" NEAR/6 ″quantitative") OR ("CT" NEAR/6 ″Scan" NEAR/6 ″quantitative") OR ("CT" NEAR/6 ″Scans" NEAR/6 ″quantitative") OR ("CT" NEAR/6 ″quantitative")) OR ("Radiography" AND "dynamic") OR (("Radiography" NEAR/6 ″dynamic") OR ("X-ray" NEAR/6 ″dynamic") OR ("Xray" NEAR/6 ″dynamic") OR ("X-rays" NEAR/6 ″dynamic") OR ("Xrays" NEAR/6 ″dynamic") OR ("roentgen" NEAR/6 ″dynamic") OR ("rontgen" NEAR/6 ″dynamic")))) OR (TS=("posterior lumbar fusion" OR "posterior lumbar fusions" OR "posterior lumbar spine fusion" OR "posterior lumbar spine fusions" OR "posterior lumbosacral fusion" OR "posterior lumbar interbody fusion" OR "posterior lumbar interbody fusions" OR "posterior lumbar inter body fusion" OR "posterolateral lumbar fusion" OR "posterolateral lumbar fusions" OR "posterolateral lumbar spine fusion" OR "posterolateral lumbar spine fusions" OR "posterolateral lumbosacral fusions" OR "posterolateral lumbosacral fusion" OR "posterolateral lumbar interbody fusion" OR "posterior lumbosacral fusions" OR "posterior lumbar inter body fusions" OR "posterolateral lumbar interbody fusions" OR "posterolateral lumbar inter body fusion" OR "posterolateral lumbar inter body fusions" OR (("posterior" NEAR/6 ″lumbar" NEAR/6 ″fusion") OR ("posterior" NEAR/6 ″lumbar" NEAR/6 ″fusions") OR ("posterior" NEAR/6 ″lumbar" NEAR/6 ″spine" NEAR/6 ″fusion") OR ("posterior" NEAR/6 ″lumbar" NEAR/6 ″spine" NEAR/6 ″fusions") OR ("posterior" NEAR/6 ″lumbosacral" NEAR/6 ″fusions") OR ("posterior" NEAR/6 ″lumbosacral" NEAR/6 ″fusion") OR ("posterior" NEAR/6 ″lumbar" NEAR/6 ″interbody" NEAR/6 ″fusion") OR ("posterior" NEAR/6 ″lumbar" NEAR/6 ″interbody" NEAR/6 ″fusions") OR ("posterior" NEAR/6 ″lumbar" NEAR/6 ″inter" NEAR/6 ″body" NEAR/6 ″fusion") OR ("posterior" NEAR/6 ″lumbar" NEAR/6 ″inter" NEAR/6 ″body" NEAR/6 ″fusions") OR ("posterolateral" NEAR/6 ″lumbar" NEAR/6 ″fusion") OR ("posterolateral" NEAR/6 ″lumbar" NEAR/6 ″fusions") OR ("posterolateral" NEAR/6 ″lumbar" NEAR/6 ″spine" NEAR/6 ″fusion") OR ("posterolateral" NEAR/6 ″lumbar" NEAR/6 ″spine" NEAR/6 ″fusions") OR ("posterolateral" NEAR/6 ″lumbosacral" NEAR/6 ″fusions") OR ("posterolateral" NEAR/6 ″lumbosacral" NEAR/6 ″fusion") OR ("posterolateral" NEAR/6 ″lumbar" NEAR/6 ″interbody" NEAR/6 ″fusion") OR ("posterolateral" NEAR/6 ″lumbar" NEAR/6 ″interbody" NEAR/6 ″fusions") OR ("posterolateral" NEAR/6 ″lumbar" NEAR/6 ″inter" NEAR/6 ″body" NEAR/6 ″fusion") OR ("posterolateral" NEAR/6 ″lumbar" NEAR/6 ″inter" NEAR/6 ″body" NEAR/6 ″fusions")) OR "PLIF") AND TI=("Computer Assisted Tomography" OR "CAT Scan" OR "CAT Scans" OR "Computed Tomogra∗" OR "Computed Tomography" OR "Computer Assisted Tomogra∗" OR "Computer Assisted Tomography" OR "Computer Tomogra∗" OR "Computer Tomography" OR "Computerised Axial Tomogra∗" OR "Computerised Axial Tomography" OR "Computerised Tomogra∗" OR "Computerised Tomography" OR "Computerized Axial Tomogra∗" OR "Computerized Axial Tomography" OR "Computerized Tomogra∗" OR "Computerized Tomography" OR "CT Scan" OR "CT Scans" OR "CT" OR "Radiography" OR "Radiography" OR "Radiogra∗" OR "X-ray" OR "Xray" OR "X-rays" OR "Xrays" OR "roentgen∗" OR "rontgen∗" OR "radiologic assessment" OR "radiologic assessments" OR "radiological assessment" OR "radiological assessments")) OR (TI=("posterior lumbar fusion" OR "posterior lumbar fusions" OR "posterior lumbar spine fusion" OR "posterior lumbar spine fusions" OR "posterior lumbosacral fusion" OR "posterior lumbar interbody fusion" OR "posterior lumbar interbody fusions" OR "posterior lumbar inter body fusion" OR "posterolateral lumbar fusion" OR "posterolateral lumbar fusions" OR "posterolateral lumbar spine fusion" OR "posterolateral lumbar spine fusions" OR "posterolateral lumbosacral fusions" OR "posterolateral lumbosacral fusion" OR "posterolateral lumbar interbody fusion" OR "posterior lumbosacral fusions" OR "posterior lumbar inter body fusions" OR "posterolateral lumbar interbody fusions" OR "posterolateral lumbar inter body fusion" OR "posterolateral lumbar inter body fusions" OR (("posterior" NEAR/6 ″lumbar" NEAR/6 ″fusion") OR ("posterior" NEAR/6 ″lumbar" NEAR/6 ″fusions") OR ("posterior" NEAR/6 ″lumbar" NEAR/6 ″spine" NEAR/6 ″fusion") OR ("posterior" NEAR/6 ″lumbar" NEAR/6 ″spine" NEAR/6 ″fusions") OR ("posterior" NEAR/6 ″lumbosacral" NEAR/6 ″fusions") OR ("posterior" NEAR/6 ″lumbosacral" NEAR/6 ″fusion") OR ("posterior" NEAR/6 ″lumbar" NEAR/6 ″interbody" NEAR/6 ″fusion") OR ("posterior" NEAR/6 ″lumbar" NEAR/6 ″interbody" NEAR/6 ″fusions") OR ("posterior" NEAR/6 ″lumbar" NEAR/6 ″inter" NEAR/6 ″body" NEAR/6 ″fusion") OR ("posterior" NEAR/6 ″lumbar" NEAR/6 ″inter" NEAR/6 ″body" NEAR/6 ″fusions") OR ("posterolateral" NEAR/6 ″lumbar" NEAR/6 ″fusion") OR ("posterolateral" NEAR/6 ″lumbar" NEAR/6 ″fusions") OR ("posterolateral" NEAR/6 ″lumbar" NEAR/6 ″spine" NEAR/6 ″fusion") OR ("posterolateral" NEAR/6 ″lumbar" NEAR/6 ″spine" NEAR/6 ″fusions") OR ("posterolateral" NEAR/6 ″lumbosacral" NEAR/6 ″fusions") OR ("posterolateral" NEAR/6 ″lumbosacral" NEAR/6 ″fusion") OR ("posterolateral" NEAR/6 ″lumbar" NEAR/6 ″interbody" NEAR/6 ″fusion") OR ("posterolateral" NEAR/6 ″lumbar" NEAR/6 ″interbody" NEAR/6 ″fusions") OR ("posterolateral" NEAR/6 ″lumbar" NEAR/6 ″inter" NEAR/6 ″body" NEAR/6 ″fusion") OR ("posterolateral" NEAR/6 ″lumbar" NEAR/6 ″inter" NEAR/6 ″body" NEAR/6 ″fusions")) OR "PLIF") AND TS=("Computer Assisted Tomography" OR "CAT Scan" OR "CAT Scans" OR "Computed Tomogra∗" OR "Computed Tomography" OR "Computer Assisted Tomogra∗" OR "Computer Assisted Tomography" OR "Computer Tomogra∗" OR "Computer Tomography" OR "Computerised Axial Tomogra∗" OR "Computerised Axial Tomography" OR "Computerised Tomogra∗" OR "Computerised Tomography" OR "Computerized Axial Tomogra∗" OR "Computerized Axial Tomography" OR "Computerized Tomogra∗" OR "Computerized Tomography" OR "CT Scan" OR "CT cans" OR "CT") AND TS=("Radiography" OR "Radiography" OR "Radiogra∗" OR "X-ray" OR "Xray" OR "X-rays" OR "Xrays" OR "roentgen∗" OR "rontgen∗"))) NOT TI=("veterinary" OR "rabbit" OR "rabbits" OR "animal" OR "animals" OR "mouse" OR "mice" OR "rodent" OR "rodents" OR "rat" OR "rats" OR "pig" OR "pigs" OR "porcine" OR "horse" OR "horses" OR "equine" OR "cow" OR "cows" OR "bovine" OR "goat" OR "goats" OR "sheep" OR "ovine" OR "canine" OR "dog" OR "dogs" OR "feline" OR "cat" OR "cats") NOT (TI = "Case Report" OR TI=("Case" NEAR/3 ″Report") OR AK = "case report" OR AK=("Case" NEAR/3 ″Report")) AND LA=("english" OR "dutch") NOT DT=("meeting abstract")

### Cochrane Library:

((("posterior lumbar fusion" OR "posterior lumbar fusions" OR "posterior lumbar spine fusion" OR "posterior lumbar spine fusions" OR "posterior lumbosacral fusion" OR "posterior lumbar interbody fusion" OR "posterior lumbar interbody fusions" OR "posterior lumbar inter body fusion" OR "posterolateral lumbar fusion" OR "posterolateral lumbar fusions" OR "posterolateral lumbar spine fusion" OR "posterolateral lumbar spine fusions" OR "posterolateral lumbosacral fusions" OR "posterolateral lumbosacral fusion" OR "posterolateral lumbar interbody fusion" OR "posterior lumbosacral fusions" OR "posterior lumbar inter body fusions" OR "posterolateral lumbar interbody fusions" OR "posterolateral lumbar inter body fusion" OR "posterolateral lumbar inter body fusions" OR (("posterior" NEAR/6 ″lumbar" NEAR/6 ″fusion") OR ("posterior" NEAR/6 ″lumbar" NEAR/6 ″fusions") OR ("posterior" NEAR/6 ″lumbar" NEAR/6 ″spine" NEAR/6 ″fusion") OR ("posterior" NEAR/6 ″lumbar" NEAR/6 ″spine" NEAR/6 ″fusions") OR ("posterior" NEAR/6 ″lumbosacral" NEAR/6 ″fusions") OR ("posterior" NEAR/6 ″lumbosacral" NEAR/6 ″fusion") OR ("posterior" NEAR/6 ″lumbar" NEAR/6 ″interbody" NEAR/6 ″fusion") OR ("posterior" NEAR/6 ″lumbar" NEAR/6 ″interbody" NEAR/6 ″fusions") OR ("posterior" NEAR/6 ″lumbar" NEAR/6 ″inter" NEAR/6 ″body" NEAR/6 ″fusion") OR ("posterior" NEAR/6 ″lumbar" NEAR/6 ″inter" NEAR/6 ″body" NEAR/6 ″fusions") OR ("posterolateral" NEAR/6 ″lumbar" NEAR/6 ″fusion") OR ("posterolateral" NEAR/6 ″lumbar" NEAR/6 ″fusions") OR ("posterolateral" NEAR/6 ″lumbar" NEAR/6 ″spine" NEAR/6 ″fusion") OR ("posterolateral" NEAR/6 ″lumbar" NEAR/6 ″spine" NEAR/6 ″fusions") OR ("posterolateral" NEAR/6 ″lumbosacral" NEAR/6 ″fusions") OR ("posterolateral" NEAR/6 ″lumbosacral" NEAR/6 ″fusion") OR ("posterolateral" NEAR/6 ″lumbar" NEAR/6 ″interbody" NEAR/6 ″fusion") OR ("posterolateral" NEAR/6 ″lumbar" NEAR/6 ″interbody" NEAR/6 ″fusions") OR ("posterolateral" NEAR/6 ″lumbar" NEAR/6 ″inter" NEAR/6 ″body" NEAR/6 ″fusion") OR ("posterolateral" NEAR/6 ″lumbar" NEAR/6 ″inter" NEAR/6 ″body" NEAR/6 ″fusions")) OR "PLIF"):ti,ab,kw AND (("Computer Assisted Tomography" AND "quantitative") OR "QCT" OR (("CAT" NEAR/6 ″Scan" NEAR/6 ″quantitative") OR ("CAT" NEAR/6 ″Scans" NEAR/6 ″quantitative") OR ("Computed" NEAR/6 ″Tomography" NEAR/6 ″quantitative") OR ("Computer" NEAR/6 ″Assisted" NEAR/6 ″Tomography" NEAR/6 ″quantitative") OR ("Computer" NEAR/6 ″Tomography" NEAR/6 ″quantitative") OR ("Computerised" NEAR/6 ″Axial" NEAR/6 ″Tomography" NEAR/6 ″quantitative") OR ("Computerised" NEAR/6 ″Tomography" NEAR/6 ″quantitative") OR ("Computerized" NEAR/6 ″Axial" NEAR/6 ″Tomography" NEAR/6 ″quantitative") OR ("Computerized" NEAR/6 ″Tomography" NEAR/6 ″quantitative") OR ("CT" NEAR/6 ″Scan" NEAR/6 ″quantitative") OR ("CT" NEAR/6 ″Scans" NEAR/6 ″quantitative") OR ("CT" NEAR/6 ″quantitative")) OR ("Radiography" AND "dynamic") OR (("Radiography" NEAR/6 ″dynamic") OR ("X-ray" NEAR/6 ″dynamic") OR ("Xray" NEAR/6 ″dynamic") OR ("X-rays" NEAR/6 ″dynamic") OR ("Xrays" NEAR/6 ″dynamic") OR ("roentgen" NEAR/6 ″dynamic") OR ("rontgen" NEAR/6 ″dynamic"))):ti,ab,kw) OR (("posterior lumbar fusion" OR "posterior lumbar fusions" OR "posterior lumbar spine fusion" OR "posterior lumbar spine fusions" OR "posterior lumbosacral fusion" OR "posterior lumbar interbody fusion" OR "posterior lumbar interbody fusions" OR "posterior lumbar inter body fusion" OR "posterolateral lumbar fusion" OR "posterolateral lumbar fusions" OR "posterolateral lumbar spine fusion" OR "posterolateral lumbar spine fusions" OR "posterolateral lumbosacral fusions" OR "posterolateral lumbosacral fusion" OR "posterolateral lumbar interbody fusion" OR "posterior lumbosacral fusions" OR "posterior lumbar inter body fusions" OR "posterolateral lumbar interbody fusions" OR "posterolateral lumbar inter body fusion" OR "posterolateral lumbar inter body fusions" OR (("posterior" NEAR/6 ″lumbar" NEAR/6 ″fusion") OR ("posterior" NEAR/6 ″lumbar" NEAR/6 ″fusions") OR ("posterior" NEAR/6 ″lumbar" NEAR/6 ″spine" NEAR/6 ″fusion") OR ("posterior" NEAR/6 ″lumbar" NEAR/6 ″spine" NEAR/6 ″fusions") OR ("posterior" NEAR/6 ″lumbosacral" NEAR/6 ″fusions") OR ("posterior" NEAR/6 ″lumbosacral" NEAR/6 ″fusion") OR ("posterior" NEAR/6 ″lumbar" NEAR/6 ″interbody" NEAR/6 ″fusion") OR ("posterior" NEAR/6 ″lumbar" NEAR/6 ″interbody" NEAR/6 ″fusions") OR ("posterior" NEAR/6 ″lumbar" NEAR/6 ″inter" NEAR/6 ″body" NEAR/6 ″fusion") OR ("posterior" NEAR/6 ″lumbar" NEAR/6 ″inter" NEAR/6 ″body" NEAR/6 ″fusions") OR ("posterolateral" NEAR/6 ″lumbar" NEAR/6 ″fusion") OR ("posterolateral" NEAR/6 ″lumbar" NEAR/6 ″fusions") OR ("posterolateral" NEAR/6 ″lumbar" NEAR/6 ″spine" NEAR/6 ″fusion") OR ("posterolateral" NEAR/6 ″lumbar" NEAR/6 ″spine" NEAR/6 ″fusions") OR ("posterolateral" NEAR/6 ″lumbosacral" NEAR/6 ″fusions") OR ("posterolateral" NEAR/6 ″lumbosacral" NEAR/6 ″fusion") OR ("posterolateral" NEAR/6 ″lumbar" NEAR/6 ″interbody" NEAR/6 ″fusion") OR ("posterolateral" NEAR/6 ″lumbar" NEAR/6 ″interbody" NEAR/6 ″fusions") OR ("posterolateral" NEAR/6 ″lumbar" NEAR/6 ″inter" NEAR/6 ″body" NEAR/6 ″fusion") OR ("posterolateral" NEAR/6 ″lumbar" NEAR/6 ″inter" NEAR/6 ″body" NEAR/6 ″fusions")) OR "PLIF"):ti,ab,kw AND ("Computer Assisted Tomography" OR "CAT Scan" OR "CAT Scans" OR "Computed Tomogra∗" OR "Computed Tomography" OR "Computer Assisted Tomogra∗" OR "Computer Assisted Tomography" OR "Computer Tomogra∗" OR "Computer Tomography" OR "Computerised Axial Tomogra∗" OR "Computerised Axial Tomography" OR "Computerised Tomogra∗" OR "Computerised Tomography" OR "Computerized Axial Tomogra∗" OR "Computerized Axial Tomography" OR "Computerized Tomogra∗" OR "Computerized Tomography" OR "CT Scan" OR "CT Scans" OR "CT" OR "Radiography" OR "Radiography" OR "Radiogra∗" OR "X-ray" OR "Xray" OR "X-rays" OR "Xrays" OR "roentgen∗" OR "rontgen∗" OR "radiologic assessment" OR "radiologic assessments" OR "radiological assessment" OR "radiological assessments"):ti) OR (("posterior lumbar fusion" OR "posterior lumbar fusions" OR "posterior lumbar spine fusion" OR "posterior lumbar spine fusions" OR "posterior lumbosacral fusion" OR "posterior lumbar interbody fusion" OR "posterior lumbar interbody fusions" OR "posterior lumbar inter body fusion" OR "posterolateral lumbar fusion" OR "posterolateral lumbar fusions" OR "posterolateral lumbar spine fusion" OR "posterolateral lumbar spine fusions" OR "posterolateral lumbosacral fusions" OR "posterolateral lumbosacral fusion" OR "posterolateral lumbar interbody fusion" OR "posterior lumbosacral fusions" OR "posterior lumbar inter body fusions" OR "posterolateral lumbar interbody fusions" OR "posterolateral lumbar inter body fusion" OR "posterolateral lumbar inter body fusions" OR (("posterior" NEAR/6 ″lumbar" NEAR/6 ″fusion") OR ("posterior" NEAR/6 ″lumbar" NEAR/6 ″fusions") OR ("posterior" NEAR/6 ″lumbar" NEAR/6 ″spine" NEAR/6 ″fusion") OR ("posterior" NEAR/6 ″lumbar" NEAR/6 ″spine" NEAR/6 ″fusions") OR ("posterior" NEAR/6 ″lumbosacral" NEAR/6 ″fusions") OR ("posterior" NEAR/6 ″lumbosacral" NEAR/6 ″fusion") OR ("posterior" NEAR/6 ″lumbar" NEAR/6 ″interbody" NEAR/6 ″fusion") OR ("posterior" NEAR/6 ″lumbar" NEAR/6 ″interbody" NEAR/6 ″fusions") OR ("posterior" NEAR/6 ″lumbar" NEAR/6 ″inter" NEAR/6 ″body" NEAR/6 ″fusion") OR ("posterior" NEAR/6 ″lumbar" NEAR/6 ″inter" NEAR/6 ″body" NEAR/6 ″fusions") OR ("posterolateral" NEAR/6 ″lumbar" NEAR/6 ″fusion") OR ("posterolateral" NEAR/6 ″lumbar" NEAR/6 ″fusions") OR ("posterolateral" NEAR/6 ″lumbar" NEAR/6 ″spine" NEAR/6 ″fusion") OR ("posterolateral" NEAR/6 ″lumbar" NEAR/6 ″spine" NEAR/6 ″fusions") OR ("posterolateral" NEAR/6 ″lumbosacral" NEAR/6 ″fusions") OR ("posterolateral" NEAR/6 ″lumbosacral" NEAR/6 ″fusion") OR ("posterolateral" NEAR/6 ″lumbar" NEAR/6 ″interbody" NEAR/6 ″fusion") OR ("posterolateral" NEAR/6 ″lumbar" NEAR/6 ″interbody" NEAR/6 ″fusions") OR ("posterolateral" NEAR/6 ″lumbar" NEAR/6 ″inter" NEAR/6 ″body" NEAR/6 ″fusion") OR ("posterolateral" NEAR/6 ″lumbar" NEAR/6 ″inter" NEAR/6 ″body" NEAR/6 ″fusions")) OR "PLIF"):ti AND ("Computer Assisted Tomography" OR "CAT Scan" OR "CAT Scans" OR "Computed Tomogra∗" OR "Computed Tomography" OR "Computer Assisted Tomogra∗" OR "Computer Assisted Tomography" OR "Computer Tomogra∗" OR "Computer Tomography" OR "Computerised Axial Tomogra∗" OR "Computerised Axial Tomography" OR "Computerised Tomogra∗" OR "Computerised Tomography" OR "Computerized Axial Tomogra∗" OR "Computerized Axial Tomography" OR "Computerized Tomogra∗" OR "Computerized Tomography" OR "CT Scan" OR "CT cans" OR "CT") AND ("Radiography" OR "Radiography" OR "Radiogra∗" OR "X-ray" OR "Xray" OR "X-rays" OR "Xrays" OR "roentgen∗" OR "rontgen∗"):ti,ab,kw)) NOT (conference abstract OR meeting abstract OR conference proceeding OR conference proceedings):pt.

### CINAHL:

((TX("posterior lumbar fusion" OR "posterior lumbar fusions" OR "posterior lumbar spine fusion" OR "posterior lumbar spine fusions" OR "posterior lumbosacral fusion" OR "posterior lumbar interbody fusion" OR "posterior lumbar interbody fusions" OR "posterior lumbar inter body fusion" OR "posterolateral lumbar fusion" OR "posterolateral lumbar fusions" OR "posterolateral lumbar spine fusion" OR "posterolateral lumbar spine fusions" OR "posterolateral lumbosacral fusions" OR "posterolateral lumbosacral fusion" OR "posterolateral lumbar interbody fusion" OR "posterior lumbosacral fusions" OR "posterior lumbar inter body fusions" OR "posterolateral lumbar interbody fusions" OR "posterolateral lumbar inter body fusion" OR "posterolateral lumbar inter body fusions" OR (("posterior" NEAR/6 ″lumbar" NEAR/6 ″fusion") OR ("posterior" NEAR/6 ″lumbar" NEAR/6 ″fusions") OR ("posterior" NEAR/6 ″lumbar" NEAR/6 ″spine" NEAR/6 ″fusion") OR ("posterior" NEAR/6 ″lumbar" NEAR/6 ″spine" NEAR/6 ″fusions") OR ("posterior" NEAR/6 ″lumbosacral" NEAR/6 ″fusions") OR ("posterior" NEAR/6 ″lumbosacral" NEAR/6 ″fusion") OR ("posterior" NEAR/6 ″lumbar" NEAR/6 ″interbody" NEAR/6 ″fusion") OR ("posterior" NEAR/6 ″lumbar" NEAR/6 ″interbody" NEAR/6 ″fusions") OR ("posterior" NEAR/6 ″lumbar" NEAR/6 ″inter" NEAR/6 ″body" NEAR/6 ″fusion") OR ("posterior" NEAR/6 ″lumbar" NEAR/6 ″inter" NEAR/6 ″body" NEAR/6 ″fusions") OR ("posterolateral" NEAR/6 ″lumbar" NEAR/6 ″fusion") OR ("posterolateral" NEAR/6 ″lumbar" NEAR/6 ″fusions") OR ("posterolateral" NEAR/6 ″lumbar" NEAR/6 ″spine" NEAR/6 ″fusion") OR ("posterolateral" NEAR/6 ″lumbar" NEAR/6 ″spine" NEAR/6 ″fusions") OR ("posterolateral" NEAR/6 ″lumbosacral" NEAR/6 ″fusions") OR ("posterolateral" NEAR/6 ″lumbosacral" NEAR/6 ″fusion") OR ("posterolateral" NEAR/6 ″lumbar" NEAR/6 ″interbody" NEAR/6 ″fusion") OR ("posterolateral" NEAR/6 ″lumbar" NEAR/6 ″interbody" NEAR/6 ″fusions") OR ("posterolateral" NEAR/6 ″lumbar" NEAR/6 ″inter" NEAR/6 ″body" NEAR/6 ″fusion") OR ("posterolateral" NEAR/6 ″lumbar" NEAR/6 ″inter" NEAR/6 ″body" NEAR/6 ″fusions")) OR "PLIF") AND TX(("Computer Assisted Tomography" AND "quantitative") OR "QCT" OR (("CAT" NEAR/6 ″Scan" NEAR/6 ″quantitative") OR ("CAT" NEAR/6 ″Scans" NEAR/6 ″quantitative") OR ("Computed" NEAR/6 ″Tomography" NEAR/6 ″quantitative") OR ("Computer" NEAR/6 ″Assisted" NEAR/6 ″Tomography" NEAR/6 ″quantitative") OR ("Computer" NEAR/6 ″Tomography" NEAR/6 ″quantitative") OR ("Computerised" NEAR/6 ″Axial" NEAR/6 ″Tomography" NEAR/6 ″quantitative") OR ("Computerised" NEAR/6 ″Tomography" NEAR/6 ″quantitative") OR ("Computerized" NEAR/6 ″Axial" NEAR/6 ″Tomography" NEAR/6 ″quantitative") OR ("Computerized" NEAR/6 ″Tomography" NEAR/6 ″quantitative") OR ("CT" NEAR/6 ″Scan" NEAR/6 ″quantitative") OR ("CT" NEAR/6 ″Scans" NEAR/6 ″quantitative") OR ("CT" NEAR/6 ″quantitative")) OR ("Radiography" AND "dynamic") OR (("Radiography" NEAR/6 ″dynamic") OR ("X-ray" NEAR/6 ″dynamic") OR ("Xray" NEAR/6 ″dynamic") OR ("X-rays" NEAR/6 ″dynamic") OR ("Xrays" NEAR/6 ″dynamic") OR ("roentgen" NEAR/6 ″dynamic") OR ("rontgen" NEAR/6 ″dynamic")))) OR (TX("posterior lumbar fusion" OR "posterior lumbar fusions" OR "posterior lumbar spine fusion" OR "posterior lumbar spine fusions" OR "posterior lumbosacral fusion" OR "posterior lumbar interbody fusion" OR "posterior lumbar interbody fusions" OR "posterior lumbar inter body fusion" OR "posterolateral lumbar fusion" OR "posterolateral lumbar fusions" OR "posterolateral lumbar spine fusion" OR "posterolateral lumbar spine fusions" OR "posterolateral lumbosacral fusions" OR "posterolateral lumbosacral fusion" OR "posterolateral lumbar interbody fusion" OR "posterior lumbosacral fusions" OR "posterior lumbar inter body fusions" OR "posterolateral lumbar interbody fusions" OR "posterolateral lumbar inter body fusion" OR "posterolateral lumbar inter body fusions" OR (("posterior" NEAR/6 ″lumbar" NEAR/6 ″fusion") OR ("posterior" NEAR/6 ″lumbar" NEAR/6 ″fusions") OR ("posterior" NEAR/6 ″lumbar" NEAR/6 ″spine" NEAR/6 ″fusion") OR ("posterior" NEAR/6 ″lumbar" NEAR/6 ″spine" NEAR/6 ″fusions") OR ("posterior" NEAR/6 ″lumbosacral" NEAR/6 ″fusions") OR ("posterior" NEAR/6 ″lumbosacral" NEAR/6 ″fusion") OR ("posterior" NEAR/6 ″lumbar" NEAR/6 ″interbody" NEAR/6 ″fusion") OR ("posterior" NEAR/6 ″lumbar" NEAR/6 ″interbody" NEAR/6 ″fusions") OR ("posterior" NEAR/6 ″lumbar" NEAR/6 ″inter" NEAR/6 ″body" NEAR/6 ″fusion") OR ("posterior" NEAR/6 ″lumbar" NEAR/6 ″inter" NEAR/6 ″body" NEAR/6 ″fusions") OR ("posterolateral" NEAR/6 ″lumbar" NEAR/6 ″fusion") OR ("posterolateral" NEAR/6 ″lumbar" NEAR/6 ″fusions") OR ("posterolateral" NEAR/6 ″lumbar" NEAR/6 ″spine" NEAR/6 ″fusion") OR ("posterolateral" NEAR/6 ″lumbar" NEAR/6 ″spine" NEAR/6 ″fusions") OR ("posterolateral" NEAR/6 ″lumbosacral" NEAR/6 ″fusions") OR ("posterolateral" NEAR/6 ″lumbosacral" NEAR/6 ″fusion") OR ("posterolateral" NEAR/6 ″lumbar" NEAR/6 ″interbody" NEAR/6 ″fusion") OR ("posterolateral" NEAR/6 ″lumbar" NEAR/6 ″interbody" NEAR/6 ″fusions") OR ("posterolateral" NEAR/6 ″lumbar" NEAR/6 ″inter" NEAR/6 ″body" NEAR/6 ″fusion") OR ("posterolateral" NEAR/6 ″lumbar" NEAR/6 ″inter" NEAR/6 ″body" NEAR/6 ″fusions")) OR "PLIF") AND TI("Computer Assisted Tomography" OR "CAT Scan" OR "CAT Scans" OR "Computed Tomogra∗" OR "Computed Tomography" OR "Computer Assisted Tomogra∗" OR "Computer Assisted Tomography" OR "Computer Tomogra∗" OR "Computer Tomography" OR "Computerised Axial Tomogra∗" OR "Computerised Axial Tomography" OR "Computerised Tomogra∗" OR "Computerised Tomography" OR "Computerized Axial Tomogra∗" OR "Computerized Axial Tomography" OR "Computerized Tomogra∗" OR "Computerized Tomography" OR "CT Scan" OR "CT Scans" OR "CT" OR "Radiography" OR "Radiography" OR "Radiogra∗" OR "X-ray" OR "Xray" OR "X-rays" OR "Xrays" OR "roentgen∗" OR "rontgen∗" OR "radiologic assessment" OR "radiologic assessments" OR "radiological assessment" OR "radiological assessments")) OR (TI("posterior lumbar fusion" OR "posterior lumbar fusions" OR "posterior lumbar spine fusion" OR "posterior lumbar spine fusions" OR "posterior lumbosacral fusion" OR "posterior lumbar interbody fusion" OR "posterior lumbar interbody fusions" OR "posterior lumbar inter body fusion" OR "posterolateral lumbar fusion" OR "posterolateral lumbar fusions" OR "posterolateral lumbar spine fusion" OR "posterolateral lumbar spine fusions" OR "posterolateral lumbosacral fusions" OR "posterolateral lumbosacral fusion" OR "posterolateral lumbar interbody fusion" OR "posterior lumbosacral fusions" OR "posterior lumbar inter body fusions" OR "posterolateral lumbar interbody fusions" OR "posterolateral lumbar inter body fusion" OR "posterolateral lumbar inter body fusions" OR (("posterior" NEAR/6 ″lumbar" NEAR/6 ″fusion") OR ("posterior" NEAR/6 ″lumbar" NEAR/6 ″fusions") OR ("posterior" NEAR/6 ″lumbar" NEAR/6 ″spine" NEAR/6 ″fusion") OR ("posterior" NEAR/6 ″lumbar" NEAR/6 ″spine" NEAR/6 ″fusions") OR ("posterior" NEAR/6 ″lumbosacral" NEAR/6 ″fusions") OR ("posterior" NEAR/6 ″lumbosacral" NEAR/6 ″fusion") OR ("posterior" NEAR/6 ″lumbar" NEAR/6 ″interbody" NEAR/6 ″fusion") OR ("posterior" NEAR/6 ″lumbar" NEAR/6 ″interbody" NEAR/6 ″fusions") OR ("posterior" NEAR/6 ″lumbar" NEAR/6 ″inter" NEAR/6 ″body" NEAR/6 ″fusion") OR ("posterior" NEAR/6 ″lumbar" NEAR/6 ″inter" NEAR/6 ″body" NEAR/6 ″fusions") OR ("posterolateral" NEAR/6 ″lumbar" NEAR/6 ″fusion") OR ("posterolateral" NEAR/6 ″lumbar" NEAR/6 ″fusions") OR ("posterolateral" NEAR/6 ″lumbar" NEAR/6 ″spine" NEAR/6 ″fusion") OR ("posterolateral" NEAR/6 ″lumbar" NEAR/6 ″spine" NEAR/6 ″fusions") OR ("posterolateral" NEAR/6 ″lumbosacral" NEAR/6 ″fusions") OR ("posterolateral" NEAR/6 ″lumbosacral" NEAR/6 ″fusion") OR ("posterolateral" NEAR/6 ″lumbar" NEAR/6 ″interbody" NEAR/6 ″fusion") OR ("posterolateral" NEAR/6 ″lumbar" NEAR/6 ″interbody" NEAR/6 ″fusions") OR ("posterolateral" NEAR/6 ″lumbar" NEAR/6 ″inter" NEAR/6 ″body" NEAR/6 ″fusion") OR ("posterolateral" NEAR/6 ″lumbar" NEAR/6 ″inter" NEAR/6 ″body" NEAR/6 ″fusions")) OR "PLIF") AND TX("Computer Assisted Tomography" OR "CAT Scan" OR "CAT Scans" OR "Computed Tomogra∗" OR "Computed Tomography" OR "Computer Assisted Tomogra∗" OR "Computer Assisted Tomography" OR "Computer Tomogra∗" OR "Computer Tomography" OR "Computerised Axial Tomogra∗" OR "Computerised Axial Tomography" OR "Computerised Tomogra∗" OR "Computerised Tomography" OR "Computerized Axial Tomogra∗" OR "Computerized Axial Tomography" OR "Computerized Tomogra∗" OR "Computerized Tomography" OR "CT Scan" OR "CT cans" OR "CT") AND TX("Radiography" OR "Radiography" OR "Radiogra∗" OR "X-ray" OR "Xray" OR "X-rays" OR "Xrays" OR "roentgen∗" OR "rontgen∗"))) NOT TI("veterinary" OR "rabbit" OR "rabbits" OR "animal" OR "animals" OR "mouse" OR "mice" OR "rodent" OR "rodents" OR "rat" OR "rats" OR "pig" OR "pigs" OR "porcine" OR "horse" OR "horses" OR "equine" OR "cow" OR "cows" OR "bovine" OR "goat" OR "goats" OR "sheep" OR "ovine" OR "canine" OR "dog" OR "dogs" OR "feline" OR "cat" OR "cats") NOT (TI"Case Report" OR TI("Case" NEAR/3 ″Report") OR SU"case report" OR SU("Case" NEAR/3 ″Report")) AND LA=("english" OR "dutch") NOT DT=("meeting abstract")
